# A microfluidic chip carrier including temperature control and perfusion system for long-term cell imaging

**DOI:** 10.1016/j.ohx.2021.e00245

**Published:** 2021-11-06

**Authors:** Federico Cantoni, Gabriel Werr, Laurent Barbe, Ana Maria Porras, Maria Tenje

**Affiliations:** Department of Materials Science and Engineering, Science for Life Laboratory, Uppsala University, Uppsala, Sweden

**Keywords:** Microphysiological system, Long-term cell monitoring, Functional assay on-chip, Portable microfluidics

## Abstract

Microfluidic devices are widely used for biomedical applications but there is still a lack of affordable, reliable and user-friendly systems for transferring microfluidic chips from an incubator to a microscope while maintaining physiological conditions when performing microscopy. The presented carrier represents a cost-effective option for sustaining environmental conditions of microfluidic chips in combination with minimizing the device manipulation required for reagent injection, media exchange or sample collection. The carrier, which has the outer dimension of a standard well plate size, contains an integrated perfusion system that can recirculate the media using piezo pumps, operated in either continuous or intermittent modes (50–1000 µl/min). Furthermore, a film resistive heater made from 37 µm-thick copper wires, including temperature feedback control, was used to maintain the microfluidic chip temperature at 37 °C when outside the incubator. The heater characterisation showed a uniform temperature distribution along the chip channel for perfusion flow rates up to 10 µl/min. To demonstrate the feasibility of our platform for long term cell culture monitoring, mouse brain endothelial cells (bEnd.3) were repeatedly monitored for a period of 10 days, demonstrating a system with both the versatility and the potential for long imaging in microphysiological system cell cultures.

## Graphical abstract

**Figure:**
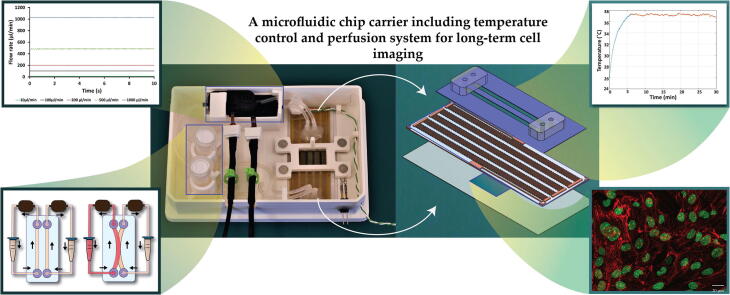

**Specifications table:** 

Hardware name	*Incubator-microscope carrier*
Subject area	• Engineering and Material Science • Biological science
Hardware type	• *Imaging tools* • *Biological sample handling and preparation*
Closest commercial analog	*Stage top incubator*
Open Source License	CC-By Attribution 4.0 International
Cost of Hardware	*Approximate cost of hardware: 630 euro*
Source File Repository	*https://doi.org/10.17605/OSF.IO/CJT8A*

## Hardware in context

In-vitro cell culture provides an invaluable instrument for biomedical studies [1]. Recently, there has been an increasing demand for such systems offering multiplexed and high-throughput analysis to obtain in-depth biological insights and to advance preclinical drug development [2].

Among the different technologies, microfluidics-based systems, so-called microphysiological systems or organ-on-chip systems, have emerged owing to the intrinsic benefits of microfluidics such as precise fluid control, reduced consumption of reagents, high throughput, and versatility [3]. Together, these features enable the *in vivo* environment to be recapitulated for biological investigations and drug screening [2], [4]. A challenge with using microphysiological systems arises when extracting information from the cells to map e.g. angiogenesis [5], proliferation [6], wound healing [7] and chemotaxis [8] as this is often done using long-term microscopy assays outside the stable environment of the incubator.

There are a few common practises aimed at reducing both exposure of the cell culture to non-optimal conditions e.g. room temperature, no cell media supply and low CO_2_, and the need to disconnect tubing, which may result in undesired pressure spikes, the introduction of air bubbles and contamination. One approach is to subdivide the full experiment into different endpoint data acquisition events, whereby separate samples are sacrificed to perform each assay. This ensures stable environmental conditions but also requires a large number of samples. Alternatively, one may use an incubator-compatible microscope for *in situ* monitoring of a single set of samples, although this significantly reduces the storage capacity of the incubator as the microscopes are rather bulky and the resolution of such microscopes is typically low.

Stage top incubators are an attractive solution for controlling the atmosphere around a microscope, guaranteeing optimal temperature, CO_2_ and humidity [9]. However, these systems are often expensive, available in limited designs and reduce the access of the microscope for other users, as the sample cannot be moved even if not used for imaging. Moreover, the platform also needs external pumps for the media supply and has limitations in terms of sample collection and reagent injection.

More recent strategies integrate miniaturized heaters directly inside the microfluidics-based cell culture platforms using thin-film deposition [10] or laser ablation [11]. The heater-cell proximity and reduced system size ensure a quick response to the environmental changes with low power consumption. The combination of such technology with micropumps on the perfusion platform leads to a compact and portable device [12], [13]. However, integrating such components on the fluidic platform restricts the chip design while increasing the cost and work required for each sample. Moreover, the integrated perfusion systems offer only a limited range of flow rates which prevents their use in high shear stress studies [14]. By transferring the heating system [15] and the perfusion onto the carrier instead of the microphysiological system, the user would benefit from a versatile tool suitable for a variety of different microfluidic platform designs.

Here, we present a versatile low-cost modular carrier with an outer dimension of a well-plate size with an integrated cell media recirculation system and heating elements compatible with an optical read-out. Microfluidic systems, with the footprint of a standard microscopy slide, can be placed inside the carrier for multiple microscopy imaging acquisitions at a constant temperature without the need to disconnect tubing when transferring the system from the incubator to the microscope. The cell media reservoirs are placed inside the carrier for easy access to allow reagents to be manually added or cell media aliquots to be collected. The cell media reservoir design avoids the introduction of air bubbles into the microfluidic system during chip manipulation.

In this work, we have characterised flow stability and investigated the temperature distribution in a mounted microfluidic system. We also demonstrate the use of the carrier with a microfluidic system in which endothelial cells are cultured to a confluent monolayer for 10 days.

## Hardware description

The carrier was made from 3D printed polylactic acid (PLA) (Ultimaker) using a commercial 3D printer (Ultimaker 2+, Ultimaker). The outer dimensions of the carrier were chosen to correspond to those of a well plate (12.7 × 8.5 × 2.7 cm^3^) to fit common optical microscope stages. The carrier surface porosity of the PLA 3D printing was sealed by depositing a 10 µm layer of Parylene C (Xinyi Chemical) via vapour deposition to prevent pathogen proliferation and simplify the sterilisation with 70% v/v ethanol washes and UV irradiation. Even though the carrier is never in contact with biological material, the Parylene C coating was chosen as an extra precaution. Alternatively, the printer can be printed in ABS (acrylonitrile–butadiene styrene) and then exposed to acetone vapours as a post-printing treatment. The vapours superficially dissolve the ABS, occluding the pores and finishing the carrier surface [16]. A recess with the size of a standard microscope slide (25 × 75 mm^2^) in the carrier bottom facilitates the alignment of the microfluidic device to the aperture (15 × 18 mm^2^) for cell imaging.

Stable temperature control at 37 °C was obtained by placing a 37-µm heating layer under the microfluidic chip. Optimal contact between the microfluidic chip and the heater was ensured by pressing the chip against the heater with a magnetic chip lock. A hold-down clamp held a thermocouple in contact with the surface of the heater. This provided a proportional-integral control (PI-loop) with feedback to regulate the heater power and maintain the set temperature of 37 °C. The PI loop was adjusted to avoid overshoot from step changes caused by e.g. rapid increases in ambient temperature during the loading of the carrier on the microscope stage. Nevertheless, a safety switch was implemented in the control software capable of turning the heaters off at a threshold overshoot of 0.5 °C and ensuring that the heater only reactivated once the temperature had fallen below the set-point (37 °C).

**Fig. 1:**
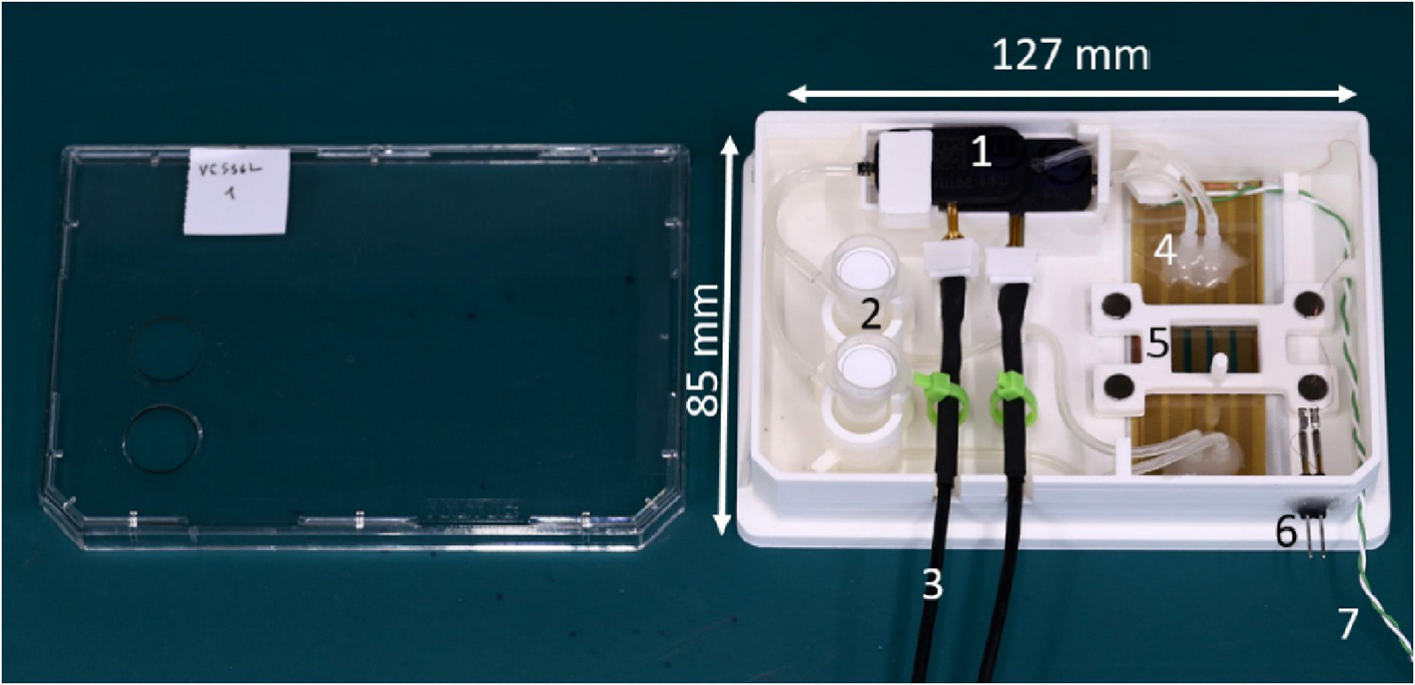
The carrier with a microfluidic chip connected to the perfusion system. 1) The two piezo pumps. 2) The two cell media reservoirs. 3) The electrical connections for the piezo-pumps. 4) The microfluidic chip. 5) The microfluidic chip lock to prevent displacement of the chip during manipulation and imaging. 6) The heater pin for the electrical connection. 7) The thermocouple.

The carrier can host up to two piezo pumps, which are arranged horizontally to protect the fragile power supply connector inside the carrier. Each pump is connected to an Eppendorf tube placed vertically inside the carrier, which acts as a cell media reservoir, Fig. 1. Eppendorf tubes from 0.5 up to 5 ml can be inserted into the carrier according to experimental needs.

The Eppendorf tube lid was replaced with a commercial porous membrane lid to allow for gas exchange. The reservoir design prevents any introduction of air bubbles and potential contaminants (e.g. microparticles or fibres) into the perfusion system while allowing easy access for manual media collection and reagent addition even inside the incubator.

**Fig. 2:**
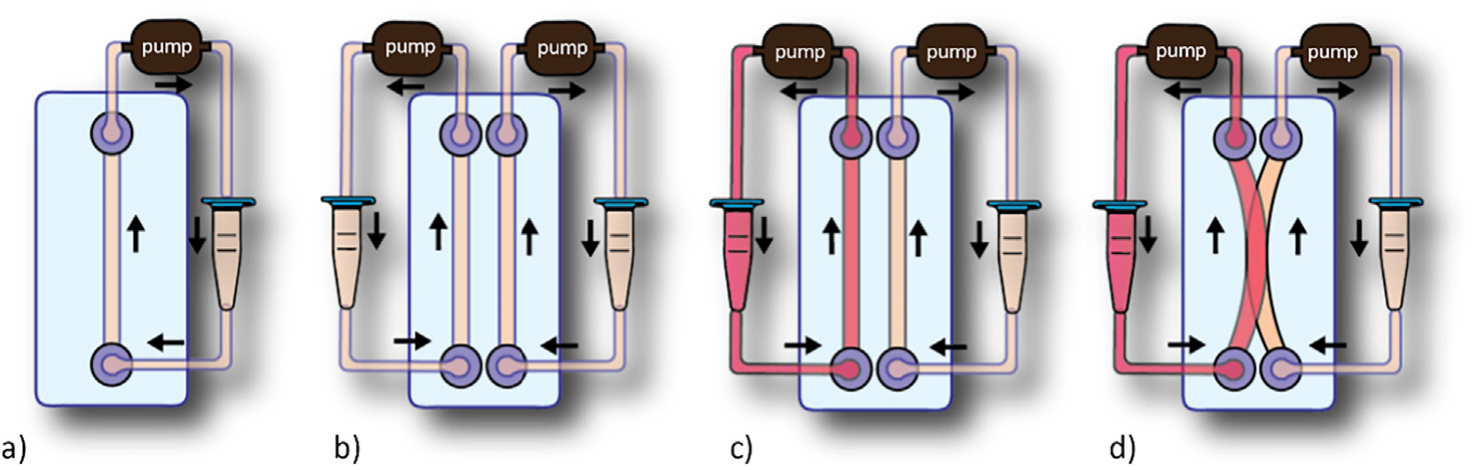
The possible perfusion configurations according to the chip design. The pump aspirates the cell media from the chip to then dispense the fluid in the reservoir in a recirculating loop. a) The single-channel perfusion. b) The double perfusion system with identical media. c) The double perfusion system with the different media and/or flow rates for each channel. d) The double perfusion system with the overlapping channels.

The system design fits several possible perfusion configurations for different chip geometries. Either a single or double channel can be connected for co-perfusion of different media or flow rates, Fig. 2.

A transparent lid with laser-cut apertures for easy access to the Eppendorf tube is used to protect the setup components from external contaminants. Both the pumps and heater are controlled and powered by a computer.

The carrier does not require the disconnection of the fluidic tubing for the transfer between the microscope and incubator. The power cord can be easily disconnected from the controlling board placed by the incubator and reconnected to another board before imaging. The heating system components (heater and thermocouple) are connected to the respective controlling board when the carrier is placed on the microscope.

The proposed carrier offers the following benefits compared to traditional cell culture on a fluidic platform:

• Reduced manipulation and contamination risk during cell media collection, reagent injection and cell imaging,
• Small cell media amount required for the recirculation (down to 0.5 ml per channel),
• User-friendly system with cost-effective manufacturing and automated cell culture,
• A versatile format for different fluidic platform sizes and designs.

## Design files

All the files for the carrier and the heater fabrication are listed in Table 1. The carrier, chip lock, pump locks and hold down clamp for the thermocouple are 3D printed with a commercial printer (Ultimaker 2+, Ultimaker) while the heater is obtained by UV lithography of a copper foil. The pump and heating system are interfaced with an Arduino board. The pump controller and respective driver are available on the pump producer website (Bartels Mikrotechnik GmbH), while the heat control hardware is available on the provided author repository.

**Table 1:** Carrier design files.

File name	File type	Open-source license	File location
Carrier	STL	CC-By Attribution 4.0 International	*https://doi.org/10.17605/OSF.IO/CJT8A*
Pump lock 1	STL	CC-By Attribution 4.0 International	*https://doi.org/10.17605/OSF.IO/CJT8A*
Pump lock 2	STL	CC-By Attribution 4.0 International	*https://doi.org/10.17605/OSF.IO/CJT8A*
Chip lock	STL	CC-By Attribution 4.0 International	*https://doi.org/10.17605/OSF.IO/CJT8A*
Thermocouple hold-down clamp	STL	CC-By Attribution 4.0 International	*https://doi.org/10.17605/OSF.IO/CJT8A*
Thermocouple holder	dxf	CC-By Attribution 4.0 International	*https://doi.org/10.17605/OSF.IO/CJT8A*
PDMS layer under the heater	dxf	CC-By Attribution 4.0 International	*https://doi.org/10.17605/OSF.IO/CJT8A*
Omni tray lid apertures	dxf	CC-By Attribution 4.0 International	*https://doi.org/10.17605/OSF.IO/CJT8A*
Heater	dxf	CC-By Attribution 4.0 International	*https://doi.org/10.17605/OSF.IO/CJT8A*

### Design files summary

• The carrier holds the microfluidic chip, piezo pumps and cell media reservoirs,
• Pump locks 1 and 2 fix the pumps to the carrier,
• The chip lock fixes the microfluidic chip to the carrier to facilitate the imaging and manipulation while enhancing the contact between the chip and the heater,
• The thermocouple hold-down clamp keeps the thermocouple against the heater to provide temperature feedback control,
• The thermocouple holder prevents direct contact between the thermocouple and the heater serpentine,
• The PDMS layer under the heater smoothen possible 3D printing defects preventing chip cracks,
• The Omni tray lid apertures on the carrier lid for easy access to the cell media reservoirs,
• The heater maintains the microfluidic chip at the physiological temperature when the carrier is outside the incubator.

## Bill of materials

Table 2 provides information regarding the fabricated and assembled mechanical components, and Table 3 lists the electronic components.

**Table 2:** Hardware components.

Designator	Component	Number	Cost per unit (Euro)	Total cost (Euro)	Source of materials
Carrier	Carrier frame	1 (50 g)	2.2	2.2	Product link
Carrier	Chip lock	1 (3 g)	0.3	0.6	Product link
Carrier	Pump locks	2 (3 g)	0.3	0.6	Product link
Carrier	Hold-down clip	1 (1 g)	0.2	0.4	Product link
Carrier	Eppendorf Tube	2 (1.5 ml)	0.07	0.14	Product link
Carrier	Silicon tubing (I.D. 1mm O.D. 2mm)	2 (15 cm)	0.22	0.2	Product link
Carrier	PEEK tubing (I.D. 0.5mm O.D. 1.2mm)	2 (1 cm)	0.028	0.056	Product link
Carrier	Tygon tubing (I.D. 1mm O.D 2mm)	1 (45 cm)	4	4	Product link
Carrier	Eppendorf tube lid with membrane	2	1	2	Product link
Carrier	Magnets	4	0.33	1.32	Product link
Carrier	Lid	1	3.28	3.28	Product link
Carrier	Needles G18	2	0.2	0.4	Product link
Heater	Heater	1	0.72	0.72	Product link

**Table 3:** Electronic components.

Designator	Component	Number	Cost per unit (Euro)	Total cost (Euro)	Source of materials
Carrier	Pump controller	1	300	503	Product link
Carrier	Pump connector	2	10	20	https://www.bartels-mikrotechnik.de/en/contact-us/
Carrier	Pump	2	30	60	Product link
Heater	Connector	2	0.04	0.08	Product link
Heater	Thermocouple	1	9.3	9.3	Product link
Heater	Heater controller	1	9	9	Product link

## Build instructions

All the components on the carrier were sterilized with ethanol 70% w/v and UV light before assembly.

### Cell media reservoir

**Fig. 3:**
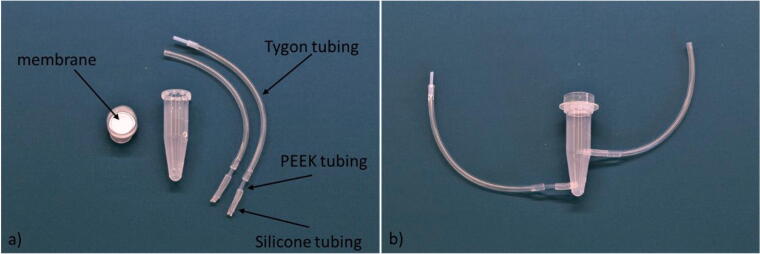
The cell media reservoir fabrication. a) The Eppendorf tube, tubing and Eppendorf tube lid with membrane. b) The fully assembled cell media reservoir with the Eppendorf membrane lid and connective tubing for the microfluidic chip and pump.

The reservoir volume can be chosen according to the requirement of the given cell culture. Here, the reservoirs consisted of 1.5 ml Eppendorf tubes with drilled holes (2 mm diameter) placed at 2 cm and 3 mm from the bottom. The chosen position of the holes ensured fibres and cell debris to settle at the bottom of the reservoir. The Eppendorf lid was removed and replaced with a membrane lid, Fig. 3a.

Tygon tubing was chosen for the fluidic connection due to the low surface roughness and reduced permeability of CO_2_. Since the plasticity of Tygon tubing prevents a tight connection to the reservoir, silicone tubing was instead chosen as the reservoir connector (I.D 1 mm O.D. 2 mm length 1 cm).

**Fig. 4:**
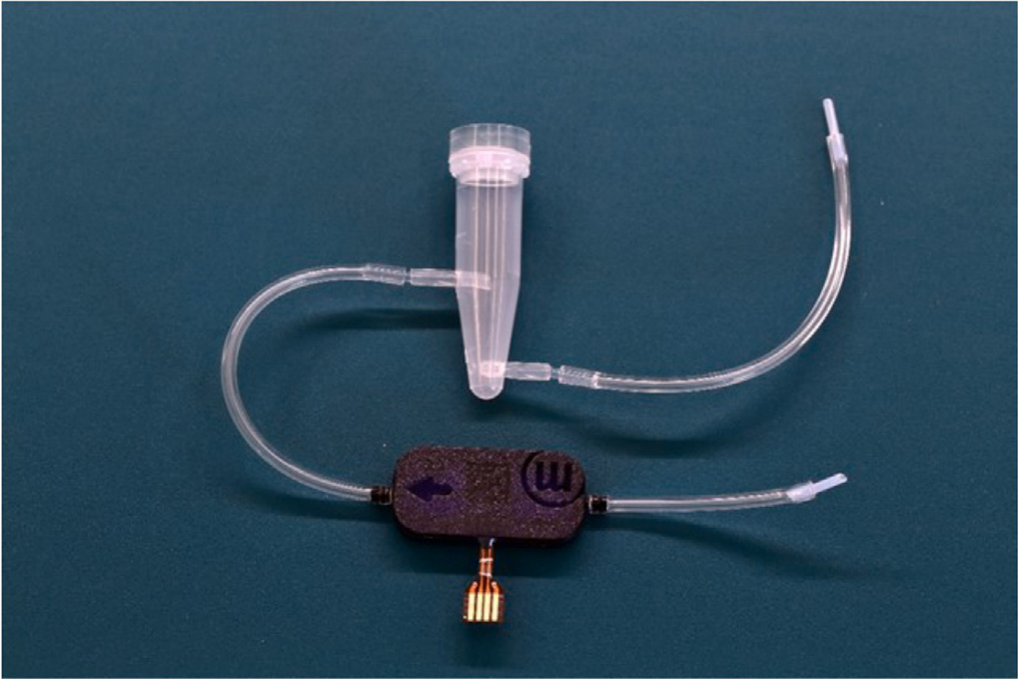
The piezo pump connected to the cell media reservoir and the tubing to the microfluidic chip.

The Tygon and silicone tubing were joined with a 2 cm long PEEK tube (I.D. 1 mm O.D 1.2 mm, VWR). The PEEK rigidity combined with the silicone softness ensured a tight seal avoiding the use of glue Fig. 3b.

The tubing dimensions used in this study are shown in Table 4.

**Table 4:** The lengths of the connecting tubing.

	inner reservoir (cm)	outer reservoir (cm)
Pump-reservoir	6.5	12
Chip-reservoir	8	10.5

Next, the pumps were connected to the respective cell media reservoir and tubing to the microfluidic chip, Fig. 4.

**Fig. 5:**
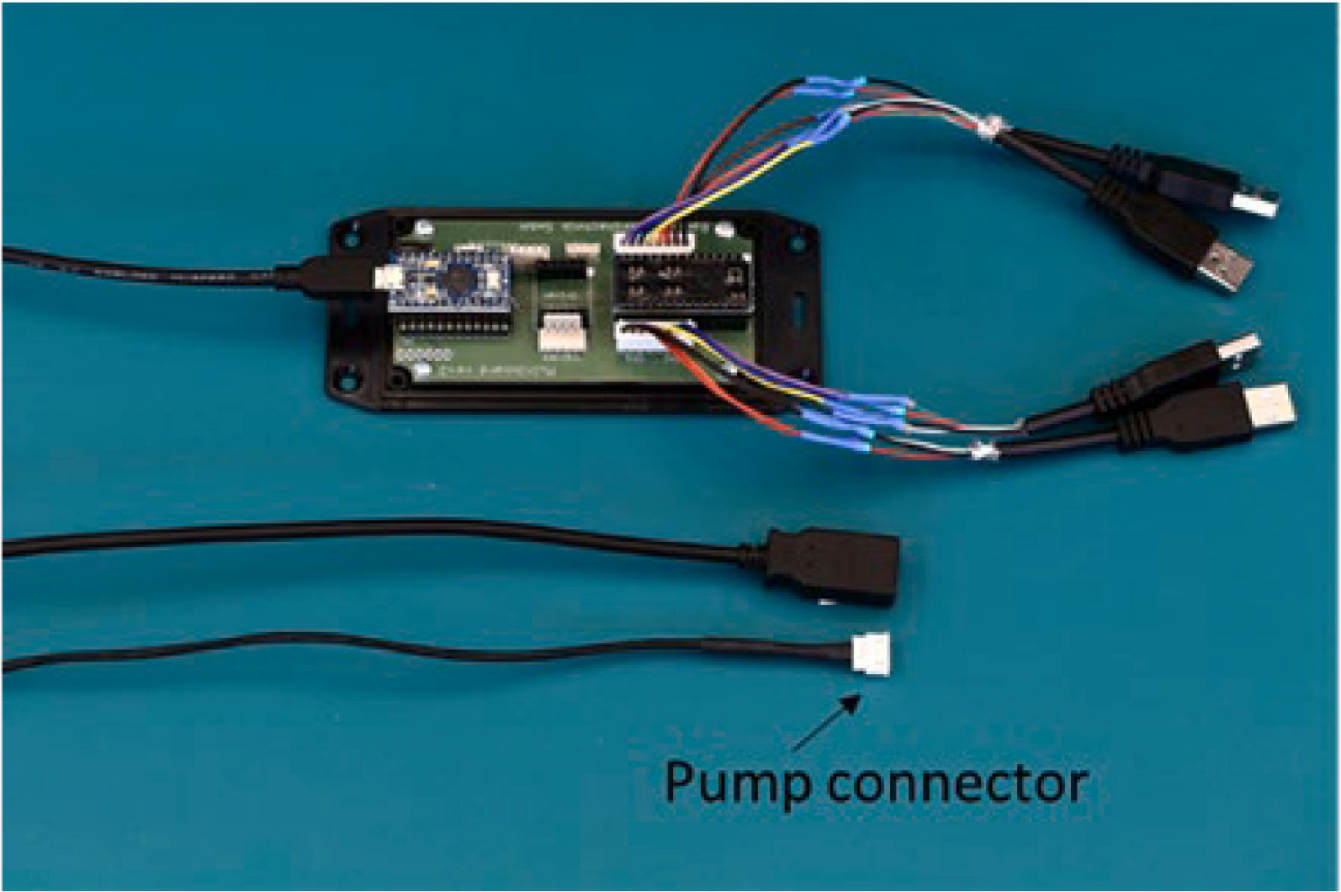
The Pump controller and the board-pump connecting cable.

### Pump controller

Since the cables connecting the controller to the pumps provided by the supplier are not designed for multiple disconnections, USB connectors were inserted onto the cable to allow for smooth disconnection of the carrier during the transfer from the incubator to the microscope, Fig. 5.

### Heater

**Fig. 6:**
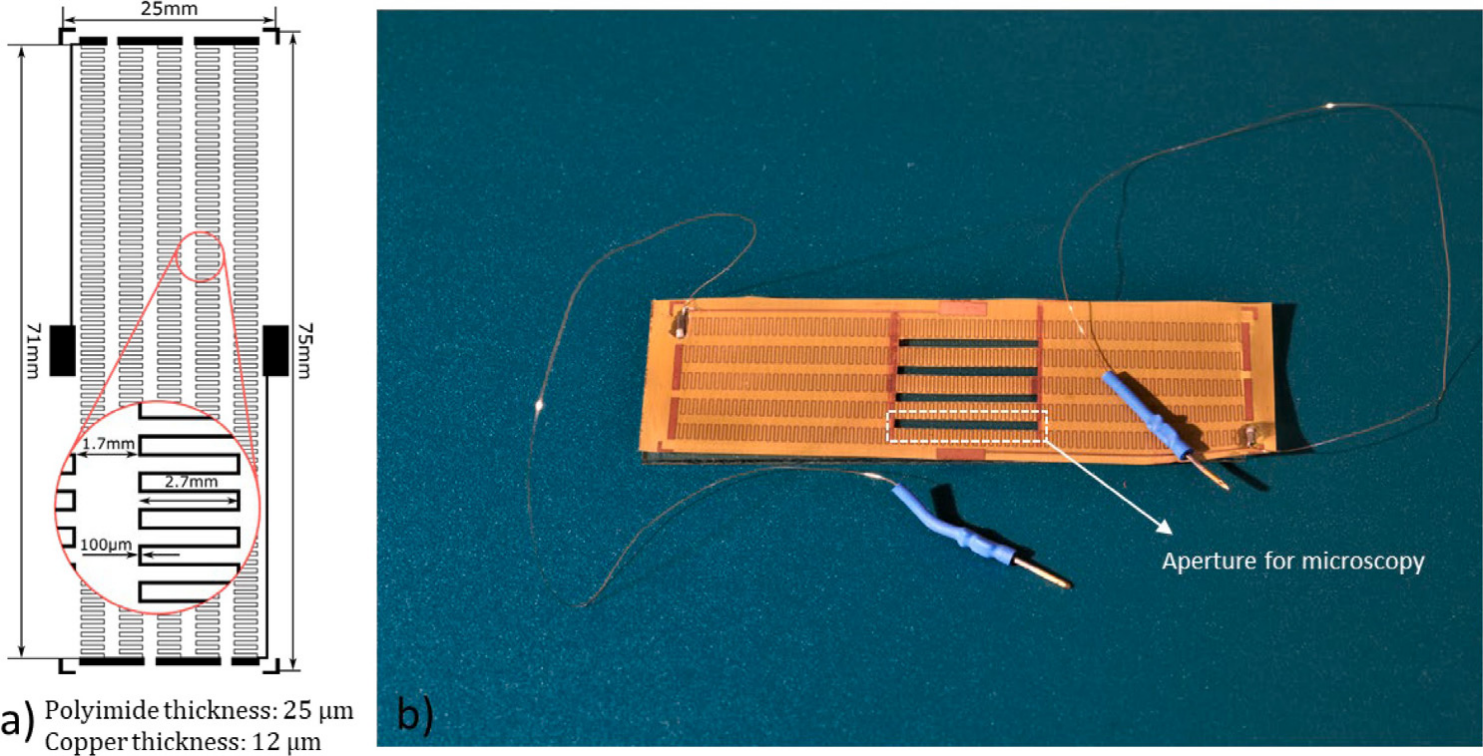
The mask for the heater fabrication with the key measurements. b) The finished heater with the connection wires attached and the aperture for microscopy cut into the polyimide.

The heater dimensions were chosen to fit a microfluidic chip with a footprint identical to that of a standard microscope slide. The heater comprises four rectangular windows for imaging of up to 4 microfluidic channels. The heater pattern was wet etched (Sodium persulfate, PROMA) into a flexible PCB substrate, consisting of 12 µm copper film and a 25 µm Polyimide backing using standard UV lithography and a 1.5 µm layer of positive photoresist (S1813). The heating elements are 71 mm long and patterned into 2.7 mm wide meanders with 0.5 mm spacing of 100 µm traces, Fig. 6. In case of no access to a cleanroom facility, the heater can also be ordered from specialised companies.

**Fig. 7:**
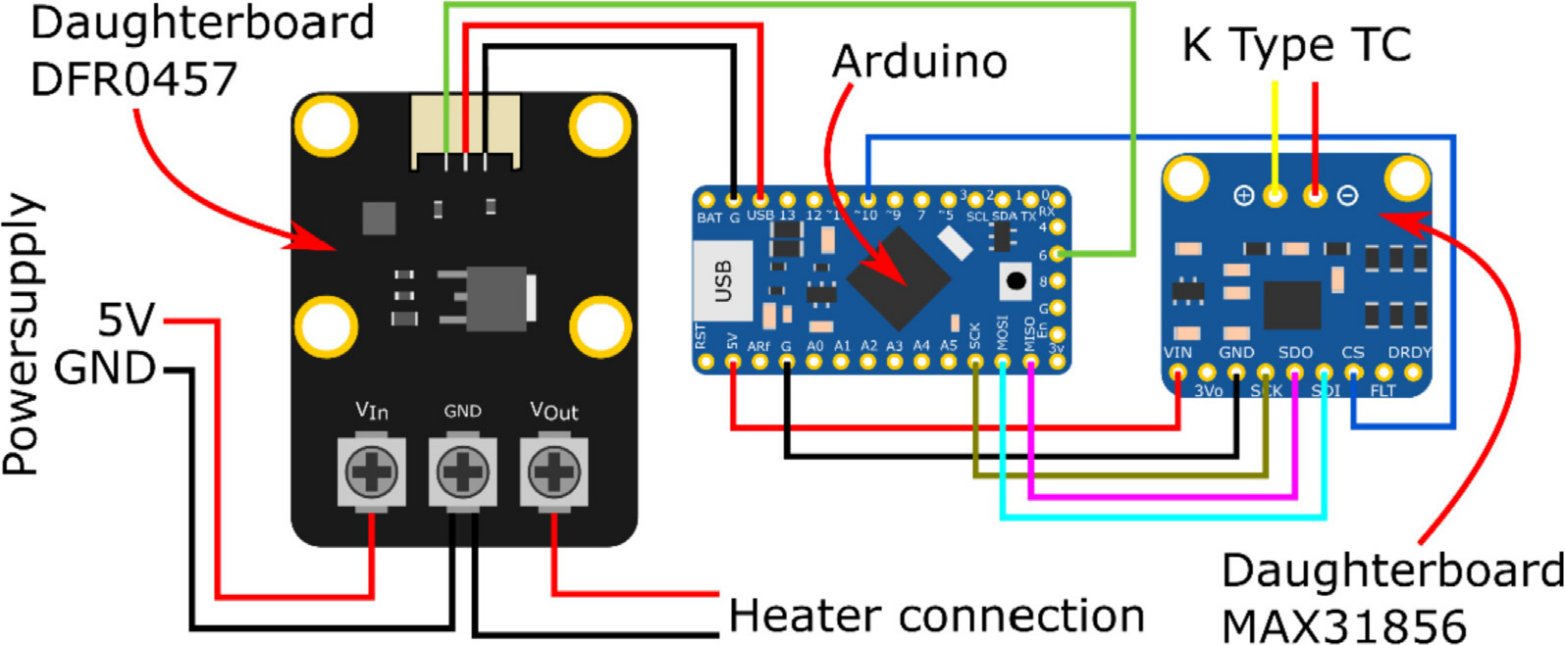
The wiring diagram for the temperature sensor and heater driver with the two daughter boards to the left and right of the Arduino.

The Polyimide backing was cut using a scalpel to open up for microscopy imaging in-between the heating elements. Wires were soldered to the contact pads of the heater. To control the temperature of the heater a PI (proportional-integral) feedback loop running on an Arduino microcontroller (ItsyBitsy 32u4 – 5 V 16 MHz, Adafruit Industries LLC, USA) was used. The temperature was measured with a K-Type thermocouple (363-0250, RS components, UK) connected to a daughterboard (Thermocouple Amplifier MAX31856, Adafruit Industries LLC, USA) and the heater power was controlled with a MOSFET, packaged in another daughterboard (DFR0457, Zhiwei Robotics Corp, China) using a pulse width modulation (PWM) signal. The heating power was 0.65 W when operating this design at 5 V. The wiring diagram can be seen in Fig. 7.

### Thermocouple holder and related hold-down clamp

**Fig. 8:**
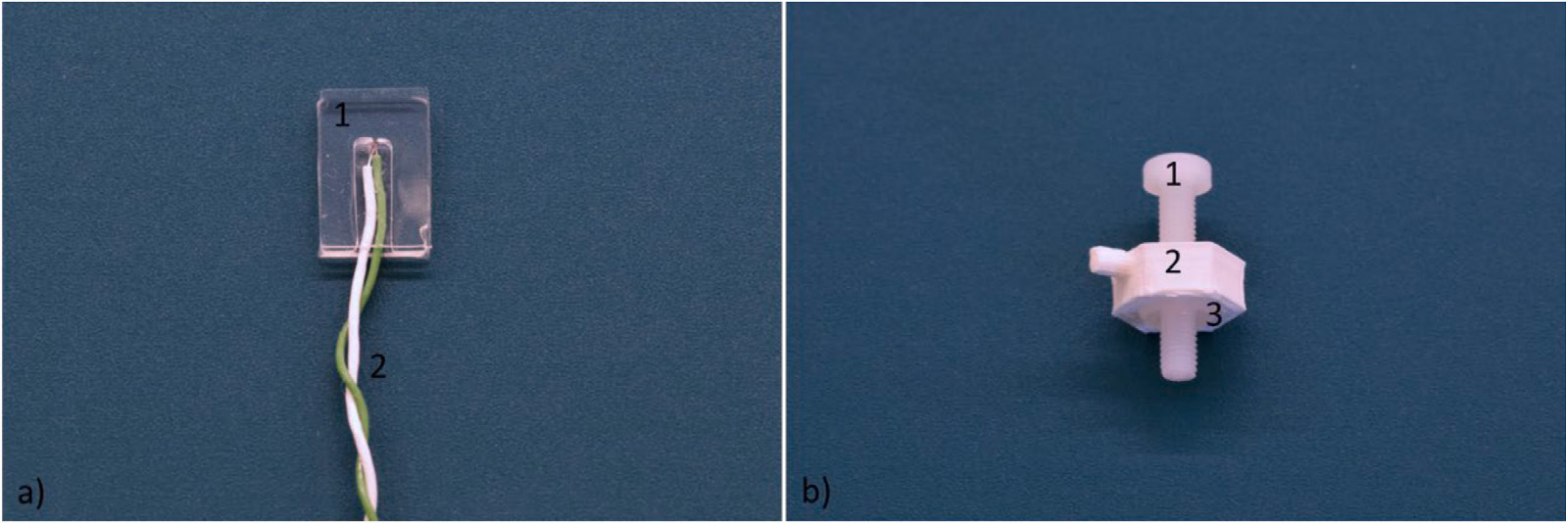
a) The thermocouple inserted in the thermocouple holder 1)The PDMS thermocouple holder. 2) The thermocouple. b) The hold-down clamp used to keep the thermocouple in touch with the heater for the temperature feedback loop. 1) The bolt. 2) The 3D printed component. 3) The nut.

The thermocouple holder was obtained by sandwiching a 250 µm thick PDMS (Polydimethylsiloxane) film, holding the thermocouple, between two layers of PDMS, 800 µm and 250 µm thick, respectively (super clear silicone sheet, Silex Silicones). To fit the thermocouple, a channel was first cut using a cutter plotter (Craft ROBO Pro, Graphtec) in the middle PDMS layer and the sandwich structure was bonded after an air plasma treatment (Model 3 Atto, Diener electronics, 200 w for 60 s). After the 3 layers of PDMS were bonded together, the thermocouple was inserted in the middle layer, Fig. 8a). The hold-down clamp maintained contact between the thermocouple and heater by pressing the thermocouple tip against the copper serpentine. The hold-down clamp consisted of a 3D printed component that holds a nut and a bolt used to keep the thermocouple in place, Fig. 8b).

### PDMS spacer between the carrier recess and the microfluidic chip

The 250 µm PDMS layer between the chip and the carrier was obtained by the cutter plotter. An aperture (15 mm *x*18 mm) was obtained to allow the central section of the microfluidic platform to be imaged.

### Carrier lid

The carrier lid was obtained by laser cutting (Östling, model: AIO G + 532 nm, shot freq: 10 kHz, power: 3.5 Watt) two holes in a commercially available lid (Nunc OmniTra, Sigma Aldrich) to provide access to the cell media reservoirs.

### Chip lock and carrier

**Fig. 9:**
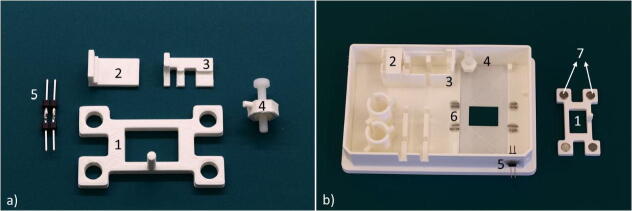
a) The carrier components and the chip lock: 1) The chip lock. 2) The pump lock 1. 3) The pump lock 2. 4) The thermocouple hold-down clamp. 5) The electrical pin connection for the heater. b) The components mounted on the carrier and the chip lock with the magnets. 1) The chip lock. 2) The pump lock 1 mounted on the carrier. 3) The pump lock 2 mounted on the carrier. 4) The thermocouple hold-down clamp mounted on the carrier. 5) The heater connectors 6) The ferromagnetic pins glued to the carrier. 7) The magnets inserted into the chip lock.

Cylindrical magnets (6 mm × 2 mm) were inserted in designated holes of the chip lock, Fig. 9a).

Ferromagnetic pins were formed by first flattening pieces of needles (G18 Sterile needles, Sterican safety needle, B. Braun) with pliers and then gluing them around the recess for the microfluidic chip. The ferromagnetic pins allow the chip lock to maintain the microfluidic platform in place during imaging and manipulations. Pump locks 1 and 2 were then glued to the carrier in the designated areas. Next, both the heater’s electrical components, obtained by soldering together two connectivity pins, and the thermocouple’s hold-down clamp, were inserted in the respective holes of the carrier walls and glued, Fig. 9b). All components were glued with silicone glue (Elastosil A07, Wacker) after air plasma oven treatment to enhance the glue adhesion (Power: 200 W, time: 5 mins, Model 3 Atto, Diener electronic).

### Microfluidic chip fabrication

The microfluidic chip design reported in this section was employed for the characterisation and validation of the carrier. However, the carrier can fit microfluidic platforms with different designs and geometries as reported in section 2.

**Fig. 10:**
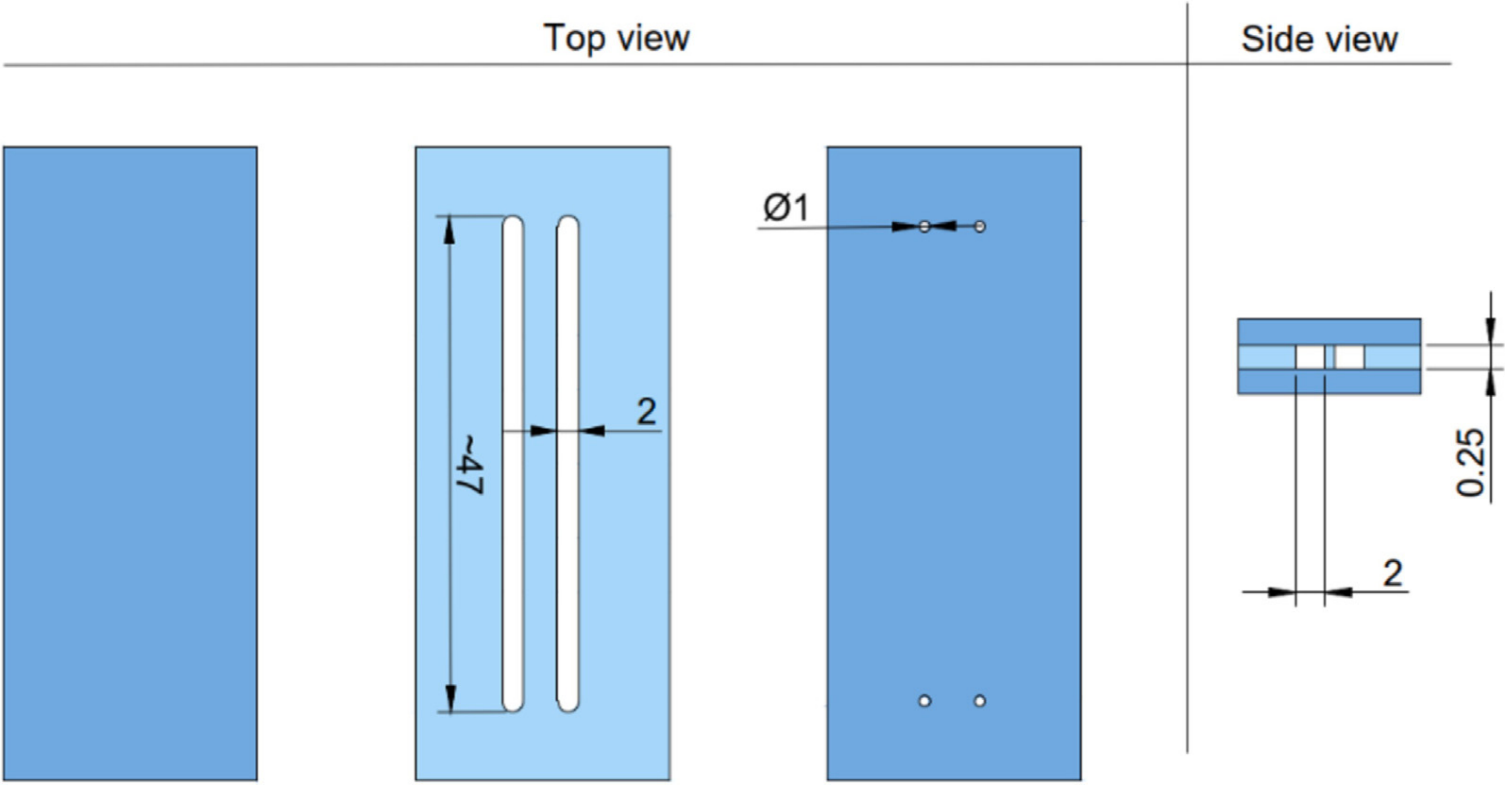
The schematic of the microfluidic chip.

The microfluidic chip used in the experiment consisted of a 250 µm-thick PDMS layer with two microfluidic channels (2 mm wide and 47 mm long) sandwiched between two 150 µm-thick coverslips (Microscope cover glasses, 24 mm × 60 mm, VWR) by air plasma (Model 3 Atto, Diener electronics, 200 w for 60 s). Silicone tubing (I.D 1 mm, O.D 3 mm length 1.5 cm) was glued on the inlet and outlet to allow the connection with the carrier perfusion system, Fig. 10.

### Assembly

**Fig. 11:**
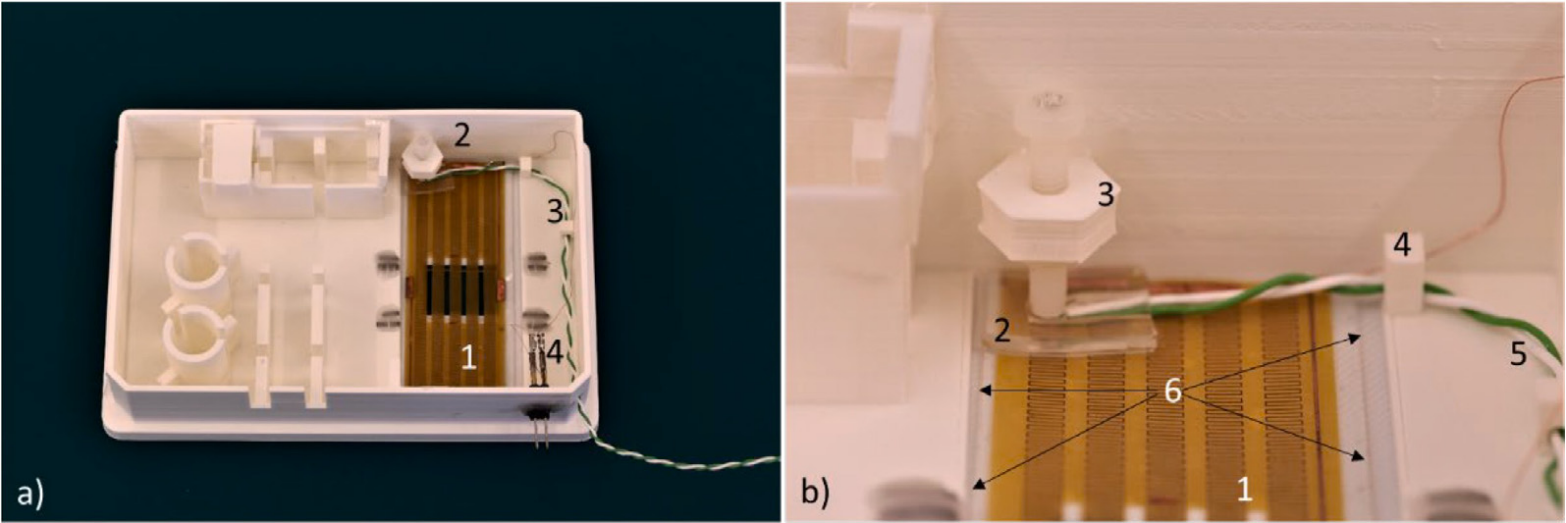
The PDMS layer, the heater and the thermocouple mounted on the carrier. 1) The heater. 2) The thermocouple hold-down clamp. 3) The thermocouple. 4) The heater connections. b) Magnification of the thermocouple hold-down clamp. 1) The heater. 2) The thermocouple holder. 3) The hold-down clamp. 4) The thermocouple guide 5) The thermocouple 6) The PDMS layer edges.

After building all the components mentioned in the previous sections, the carrier assembly was finalized as follow: First, the 250-µm PDMS layer was added on the bottom recess of the carrier, ensuring the PDMS and carrier aperture overlapped. The PDMS layer smoothed out the possible small defects due to the 3D printing. The heater was placed on top of the PDMS layer, and the electrical wires were connected to the pins. The thermocouple was pressed against the heater by tightening the screw in the hold-down clamp so that the thinnest side of the PDMS thermocouple holder was in contact with the heater. Then, the thermocouple wire was inserted in the designated guides, Fig. 11.

**Fig. 12:**
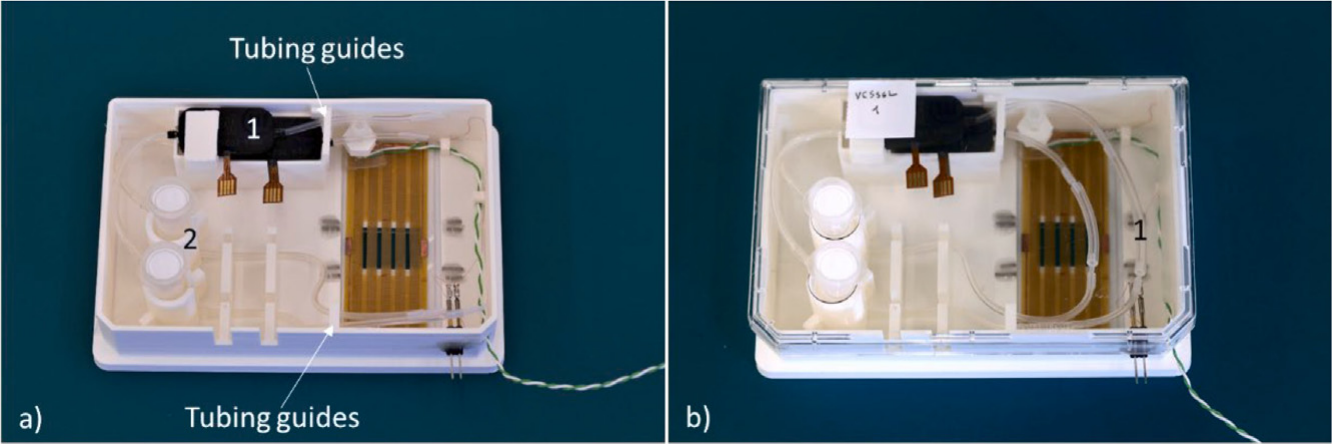
The carrier with the perfusion system mounted. 1) The piezo pumps. 2) The cell media reservoirs. b) The carrier with the protective lid.

Next, the perfusion systems were mounted on the carrier. The pumps and reservoirs were placed in the designated holders and the tubing was inserted in the respective guides, Fig. 12a). The tubing guides confine the tubes to the side of the carrier, simplifying the process of microfluidic chip manipulation. Finally, the tubes were connected in a close loop for the first cell media perfusion and the protective lid closed over the carrier, Fig. 12b). The carrier could then be stored until use.

## Operation instructions

The system operation consists of multiple stages, including thermocouple temperature offset calibration, chip loading, cell media perfusion, transfer of the system between incubator and microscope, and cell media change and/or reagent injection. For all the above-mentioned steps, the piezo pumps and heater were controlled with a computer. The piezo pump driver interface is capable of setting the working parameters of up to 4 pumps simultaneously. The frequency is limited to a common value for all pumps, whereas the voltage can be individually customised. The pump settings were inserted directly on the controller driver. A timer function embedded in the driver allowed intermittent flows to run.

### Thermocouple offset calibration

The thermocouple offset was measured by placing the carrier on the microscope stage with a microfluidic chip mounted.

**Fig. 13:**
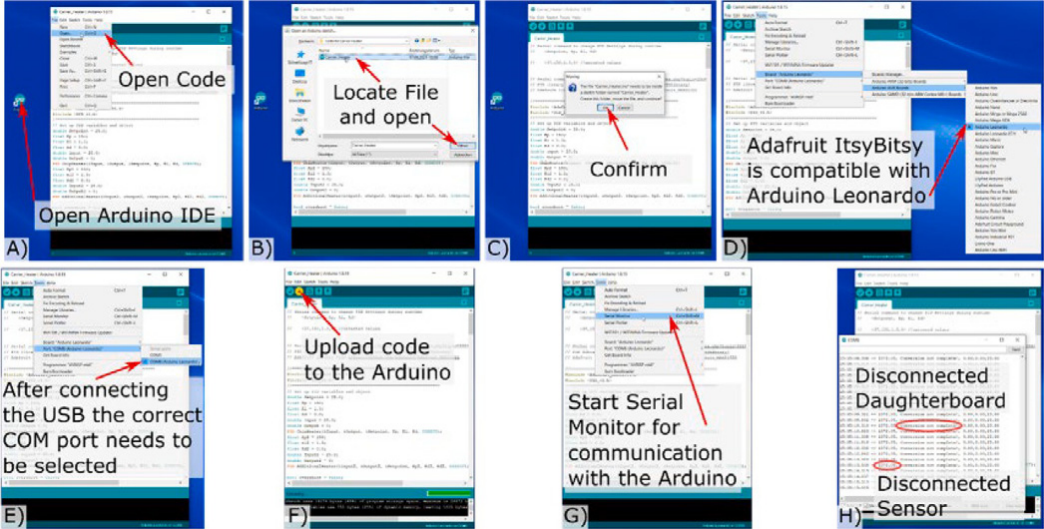
A) Opening Code in Arduino IDE, B) locating and opening code file. C) Confirm project-folder generation. D) Select Board type for programming. E) Select COM port the Arduino is connected to. F) Upload code to Arduino. G) Starting serial monitor for Data read out H) Reasons for faulty outputs I) Explanation of output columns. J) Setting of PID parameters and target Setpoint temperature.

First, a thermocouple was inserted in one of the microfluidic channels to record the reference temperature inside the channel. After mounting the chip inside the carrier, the system was placed on the microscope stage with the heater and thermocouples connected to the heating board. To operate the heater, the Arduino serial monitor was opened and a temperature thermocouple in the microfluidic chip was set to 37 °C. When the thermocouple in the microfluidic chip reached 37 °C, the temperature of the thermocouple on the heater was set as the reference temperature for the PI-loop. The heater script can be found in the project repository and the read-out from the Arduino PI-loop is shown in Fig. 13.

### Chip loading on the carrier for cell culture

**Fig. 14:**
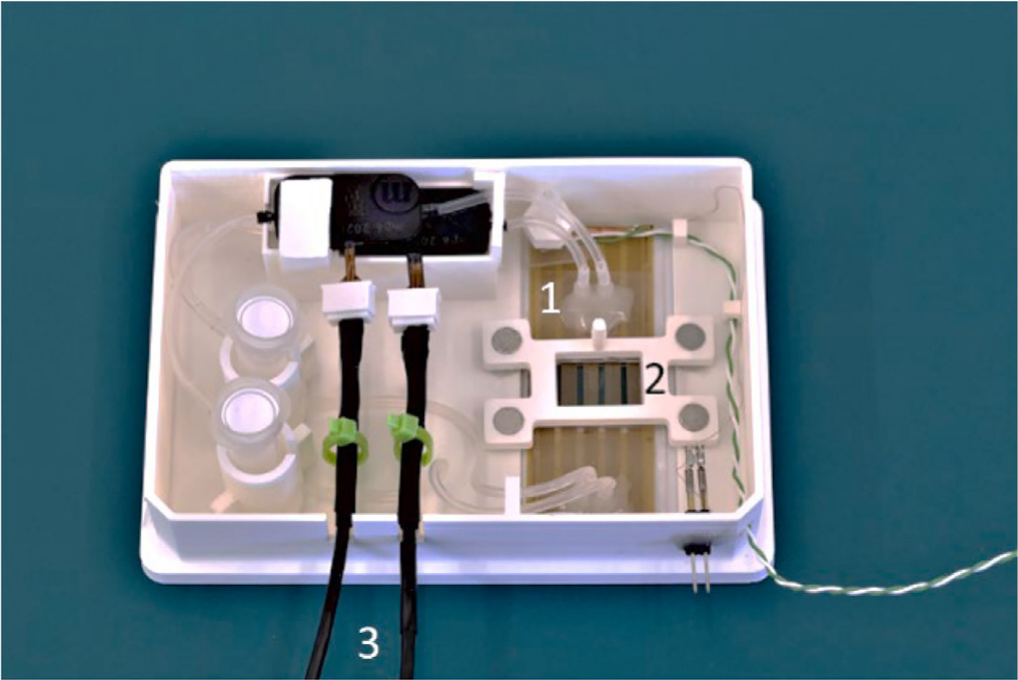
The microfluidic chip loaded on the carrier. 1) The microfluidic chip 2) The chip lock. 3) The multiboard and pumps connectors.

After calibrating the thermocouple temperature offset, the power cords were connected and fixed to the carrier with zip ties. Then, the reservoir was filled with cell media and the piezo pump switched on to fill the perfusion system. To obtain the optimal cell media pH, the carrier was placed in the incubator for at least 4 h to equilibrate the media. The carrier was removed from the incubator and, after removing the lid, the microphysiological system was mounted at the designated location. The tubing from the piezo pump and the reservoir were connected to the inlet/outlet of the chip, respectively, Fig. 14. At this step, care must be taken to avoid introducing air bubbles.

When all the tubing had been connected, the lid was closed over the carrier and placed in the incubator. Finally, the pumps were connected to the multiboard to start cell medium perfusion.

### Cell media perfusion

**Fig. 15:**
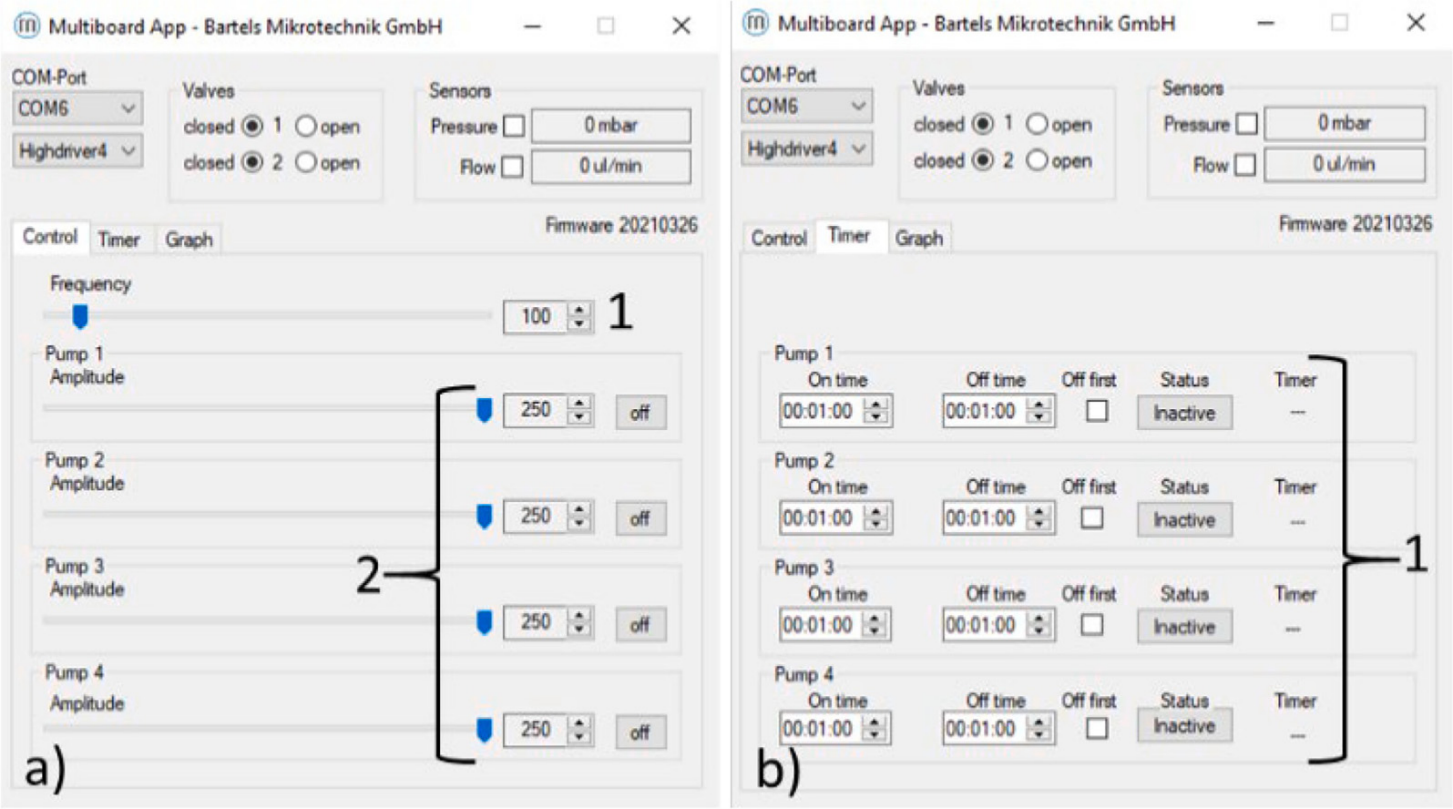
The multiboard interface. a) The control window to set the frequency and the voltage of the pumps. 1 The pump frequency. 2 the pump voltage. b) The time interval window to set the interval for intermittent perfusion. 1 The interval settings for the four pumps.

The cell media was recirculated inside the microfluidic chip. The liquid flow rate can be adjusted, stopped and started according to experimental needs, via the multiboard interface provided by the supplier, Fig. 15.

### Cell media change and/or reagent injection

The carrier design allowed the liquid to be injected and/or removed from the reservoir. This manipulation is easily performed both inside and outside the incubator due to the facile design of the inlet ports. To add/remove cell media, the piezo pump needs to be switched off to prevent air from being introduced into the perfusion system. The reservoir is then easily accessed with a pipette tip by removing the Eppendorf membrane lid. When manipulation is complete, the Eppendorf tube is closed and the piezo pumps are turned on again.

### Transfer of the sample between incubator and microscope

**Fig. 16:**
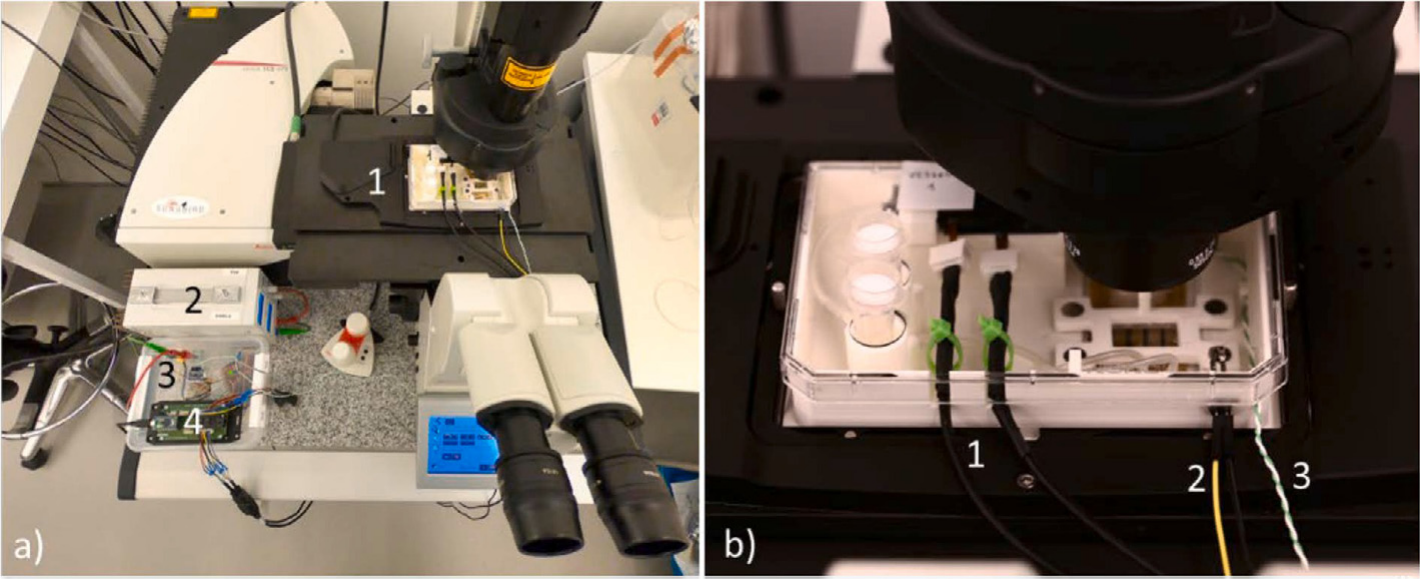
A) The carrier set-up while imaging. 1) The carrier on the microscope stage. 2) The power generator for the heater. 3) The pump controller. 4) The heater controller. b) The carrier on the microscope stage. 1) The pump power cords. 2) The heater power cord. 3) The thermocouple.

The carrier can be moved outside of the incubator by disconnecting the electrical cable connecting the pump to the board. First, Parafilm is mounted on the medium reservoir to reduce gas exchange when the carrier is moved outside of the incubator. Then, the pump is stopped by the driver and the cable is disconnected. At this point, the carrier can be moved to the microscope where the pumps are connected to a multiboard, the heater to the power supply and the thermocouple to the PI loop controller. The carrier transfer is performed without needing to disconnect any of the tubing used for media perfusion, Fig. 16.

When the carrier shall be moved back to the incubator, both the heating and pumping systems need to be disconnected. After placing the carrier back in the incubator, the Parafilm is removed and the pumps are re-connected to the multiboard to restart the perfusion.

## Characterization and validation

### Pump and perfusion system

**Fig. 17:**
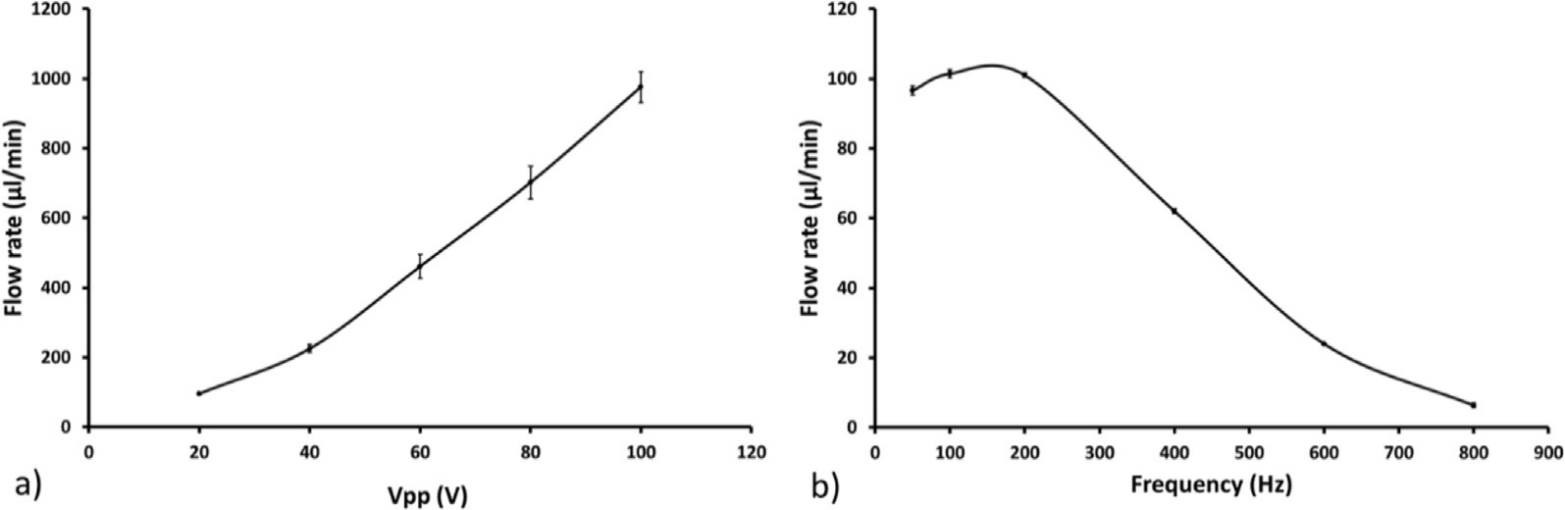
Characterization of the flow rate generated by the piezo pump. a) The flow rate in the perfusion system when applying a voltage between 20 and 100 Vpp. b) The flow rate obtained by screening the available frequency range of the pump board at 20 Vpp. Error bar from standard deviation (N = 3).

A piezoelectric pump (Bartels Mp6 pump, Bartels Mikrotechnik GmbH) was chosen for the carrier as it can sustain media recirculation at a flow rate suitable for cell culture (from 10 µl/min up to 6000 µl/min, max pressure 600 mbar, as stated by the supplier) [17]. The flow rate of the pump is set by adjusting both the input peak-to-peak voltage (V_pp_) and frequency, taking into account the system’s hydraulic resistance. The flow rate was measured for applied voltages between V_pp_ = 10–100 at a frequency of 50 Hz using a flow sensor (Sensirion SL1000, Sensirion). This measurement was also performed for frequencies between 50 and 800 Hz, Fig. 17.

**Fig. 18:**
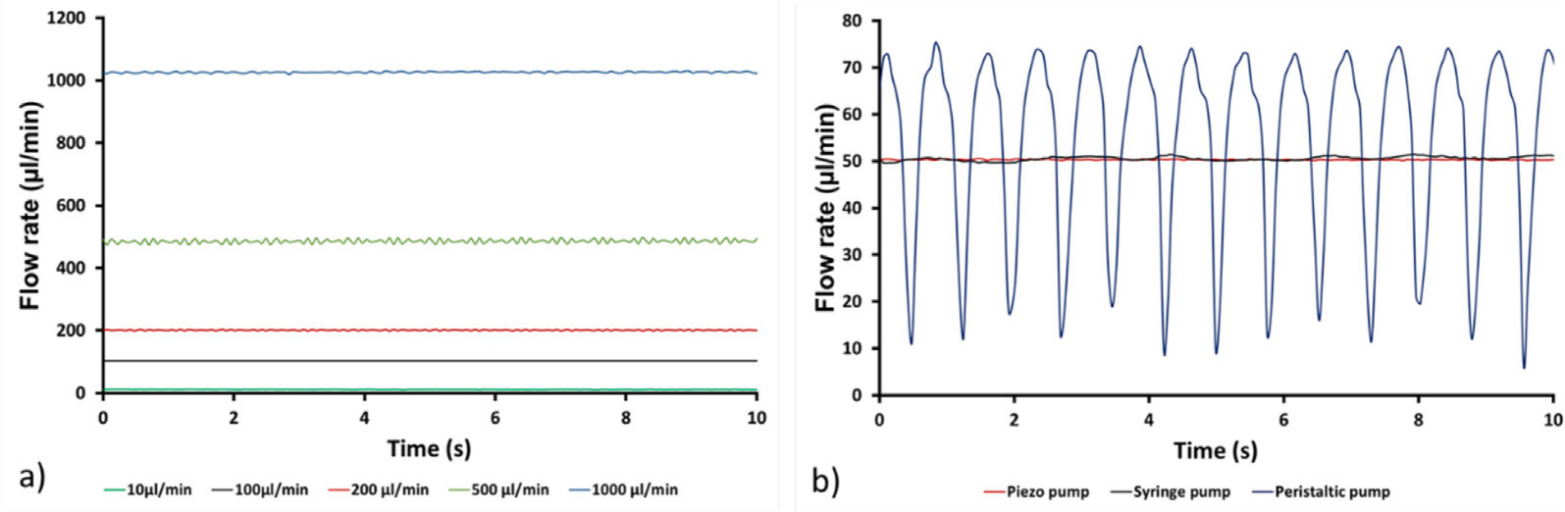
The flow rate of the piezo pump, a commercial peristaltic and syringe pump. a) The flow rate pattern of the piezo pump at 10–100-200–500 and 1000 µl/min. b) The comparison of the pulsatile behavior of a piezo pump, a peristaltic pump and a syringe pump at 50 µl/min.

The experimental data in Fig. 17a) shows that the flow rate increased as the applied voltage was increased, as expected due to the larger membrane deflections generated. Voltages below 20 Vpp did not generate sufficient pressure to create flow in the fluidic circuit. In Fig. 17 b), an increase in frequency between 50 Hz and 200 Hz did not correspond to a noticeable difference in the generated flow rate. However, frequencies above 200 Hz resulted in a decreased flow rate as the membrane deformation was no longer synchronized with the driving frequency, thus reducing the pumping capacity. To investigate the flow stability of the pumps, the flow rates of the piezo pump were measured at set flow rates of 10, 100, 200, 500 and 1000 µl/min. For comparison, the same measurements were performed with two alternative pumping systems, a syringe pump (Low-Pressure Syringe Pump Nemesys 290 N, Nemesys) and a peristaltic pump (LabV1, tubing head I.D. 0.380 mm, Shenchen) at a set flow rate of 50 µl/min. The results of these comparative studies are shown in Fig. 18.

The piezo pump displayed a continuous and stable flow rate over the whole range of the evaluated flow rates, Fig. 18a). In Fig. 18b), the comparison among the flow rates of the different pump technologies showed a similar behaviour of the piezo pump and the syringe pump, indicating uniform flow when compared with the peristaltic pump that displayed large and regular variations.

### Heater characterization

**Fig. 19:**
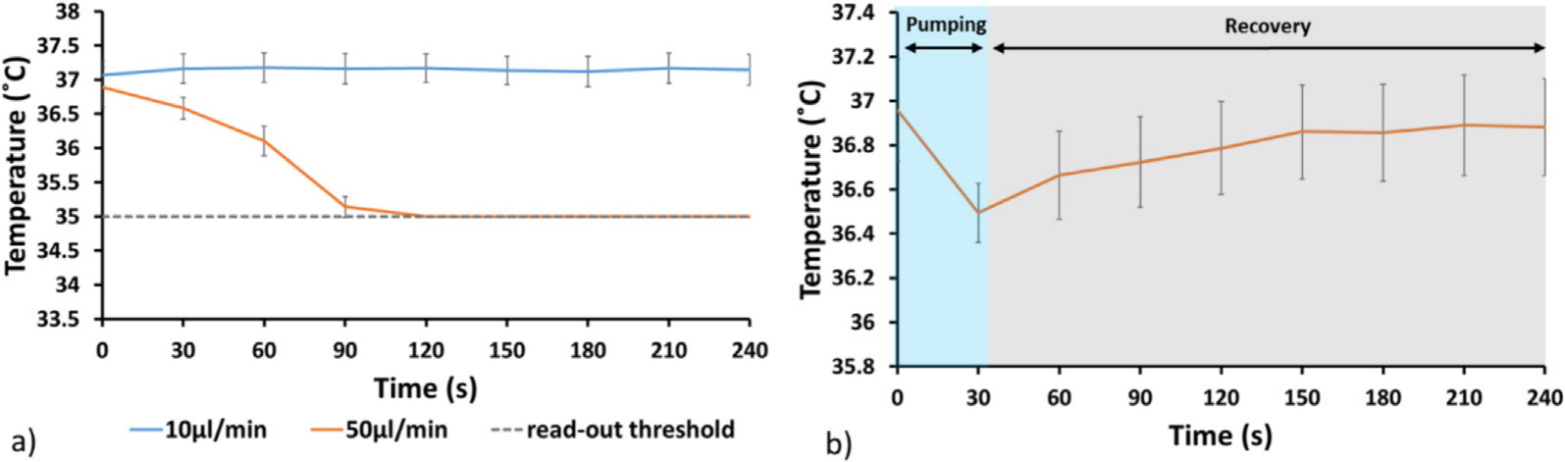
a) The temperature evolution inside the microfluidic channel flow rate at 10 and 50 µl/min. b) The temperature recovery of the heating system from a flow at 50 µl/min for 30 s. Error bar from standard deviation (N = 4).

Thermal control is essential in cell cultures as fluctuations of even just a few degrees from the ideal temperature affects the cell’s biochemical processes [18]. In the presented system, measurements of both local and total temperature distribution in the microphysiological system were carried out when the carrier was outside the incubator. To detect the local temperature distribution in the microfluidic channels, thermochromic paper (liquid crystal sheet, Edmund Optics) was used. The total temperature distribution of the PDMS/glass microfluidic chip was thermally imaged with an infrared camera. For both measurement techniques, the temperature was determined by injecting room temperature water at different flow rates; 10, 50 and 100 µl/min. Moreover, the recovery time of the heating system was tested after a flow was applied at 50 µl/min for 30 s. The perfusion time of 30 s was chosen to ensure complete turnover of the liquid inside the channel (channel volume 25 µl). Thermochromic paper strips with a temperature range of 5 °C (35–40 °C) were cut and inserted inside the channels during chip assembly to ensure an accurate local temperature evaluation. The colour change of the paper was imaged with a stereomicroscope (Nikon SMZ 745 T). The obtained image sequence was converted from colour to RGB with Cell Profiler software (version 4.0.7) and the intensity of the RGB channels was used to determine the temperature evolution over time. The paper chromatic variance within the working temperature range from 35 to 40 °C degrees was calibrated on a hotplate. The results are reported in Fig. 19.

Fig. 19a) shows the temperature mapping of the system. From these measurements the best performance i.e. the flow rate that maintained a suitable cell culture temperature, was obtained at 10 µl/min. However, the use of a 10 µl/min flow rate results in increased sensitivity to small hydraulic resistance perturbations in the system due to the low working pump power. These perturbations may be the result of bubbles, cell clumps, trapped fibres, or bent tubing. For flow rates of 50 and 100 µl/min, the temperature decreased below 35 °C (lower limit of the thermochromic paper) after 120 s and before 30 s (not shown on the graph) respectively, and we concluded that these are not viable options. For this reason, we evaluated the capacity of the system to recover to 37 °C after perfusing at a 50 µl/min flow rate for 30 s, Fig. 19b). This was accomplished after only 4 min and hence we chose to operate the system, when on the microscope, with an intermittent flow profile of 50 µl/min at 30 s intervals every hour.

**Fig. 20:**
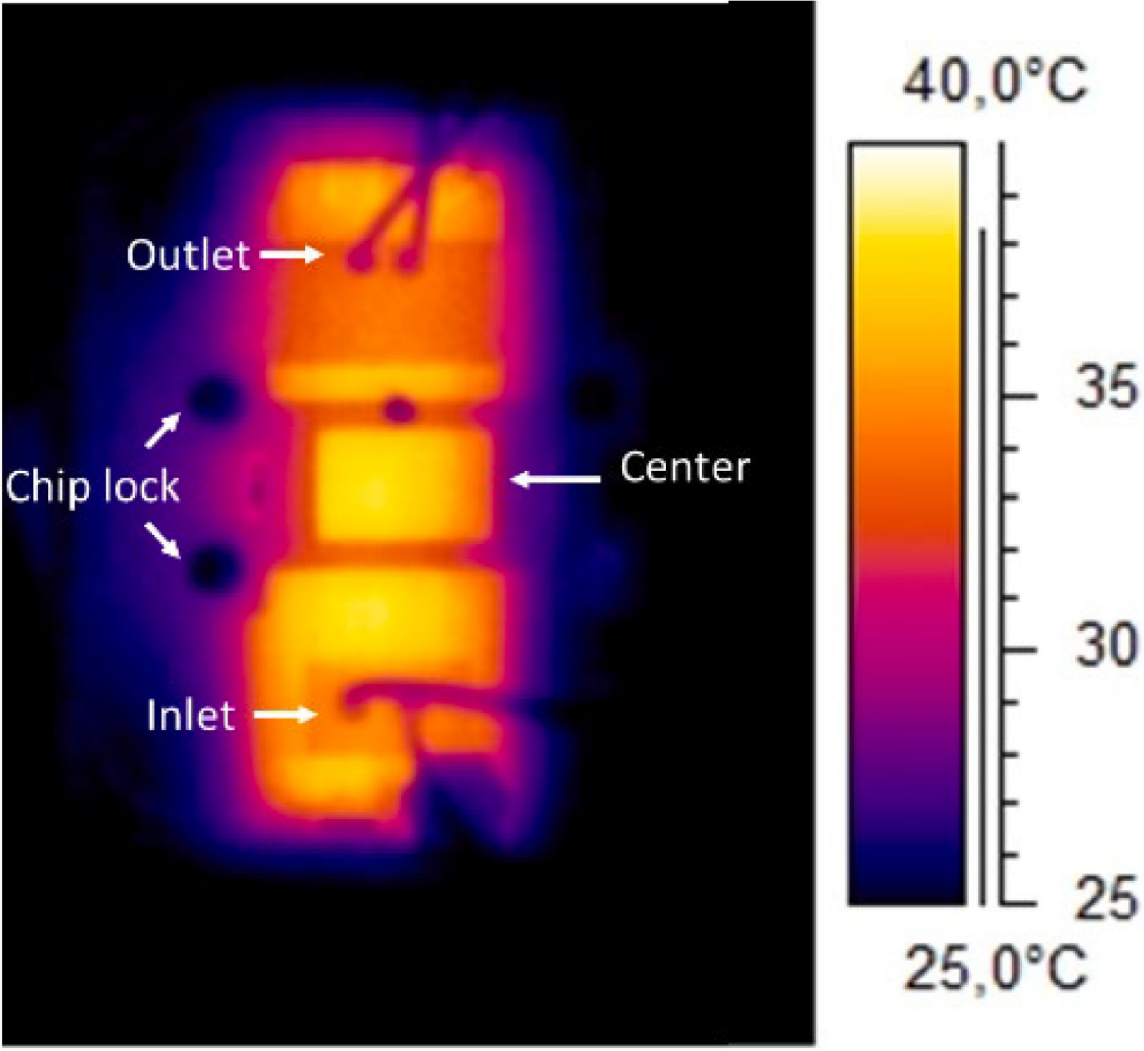
The temperature distribution after the recovery of the cell media turnover inside the microfluidic chip.

To investigate the temperature uniformity along the channel, an IR camera (A40, Teledyne FLIR) was employed. Despite its lower temperature resolution, the IR camera provided a good overview of any temperature gradients in the chip compared to the thermochromic paper. The temperature close to the inlet and at the centre of the chip was monitored when a flow of 50 µl/min was applied for 30 s. A temperature difference within 0.5 °C was detected between the two regions during the experiment, Fig. 20.

### Heater evaluation with the water objective

**Fig. 21:**
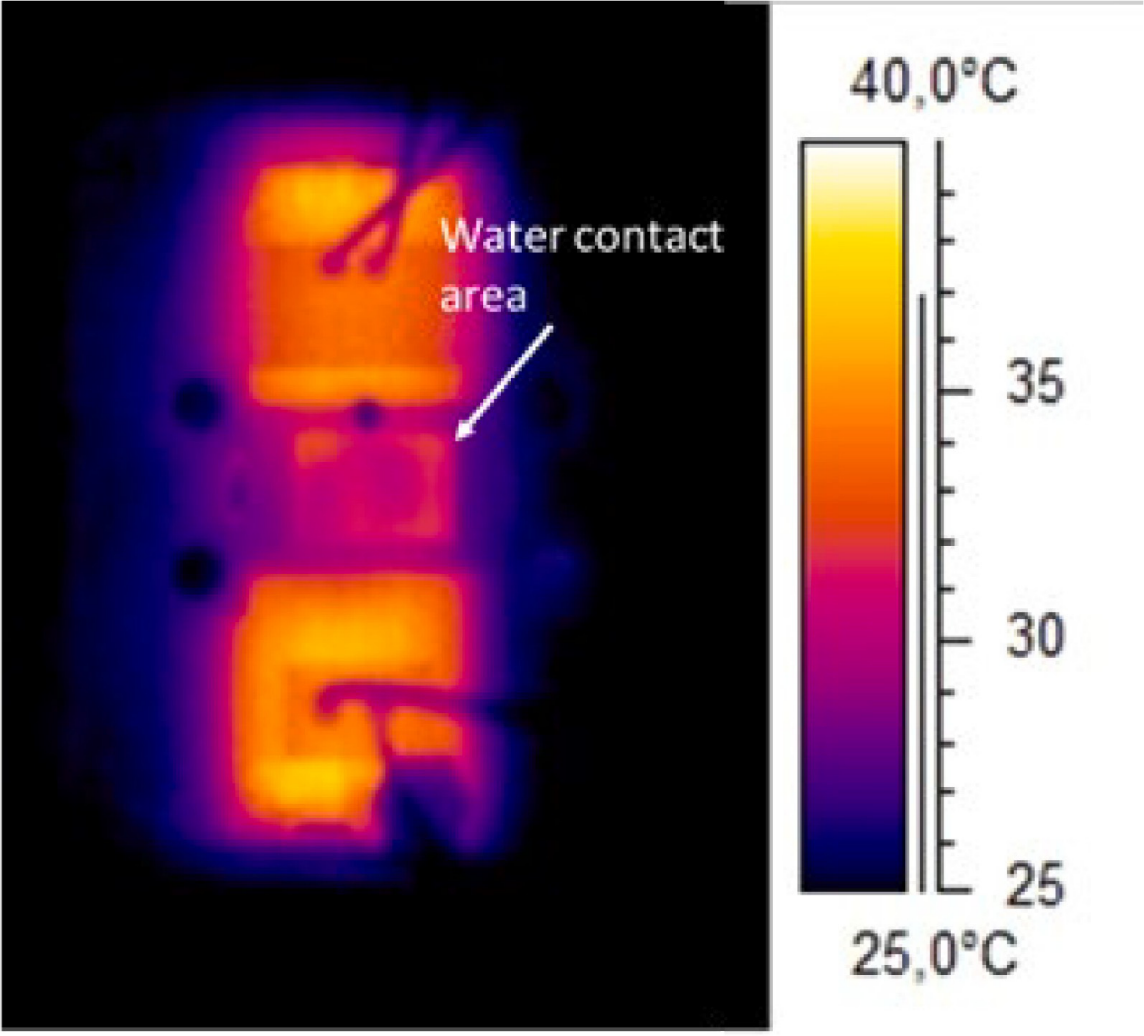
The IR camera image showing the heat sink effect of the water objective on the microfluidic chip.

For high-resolution imaging, a water/oil objective is often used. The liquid in contact with the sample and the objective works as a thermal bridge, causing cooling of the sample during image acquisition [19]. The capability of the heater to compensate for this heat sink was evaluated by placing a water objective under the system and monitoring the temperature evolution with the IR camera, Fig. 21.

In Fig. 21, the IR picture shows the local effect of the objective where the temperature of the area in contact with the objective decreased to ∼32 °C in less than 1 min, thus creating a thermal gradient between the imaging area and the remaining section of the chip that the heating system did not manage to compensate for. This implies that the carrier is not suitable for imaging that requires a water objective unless a heated collar is wrapped around the objective.

### pH evolution in the cell media reservoir

**Fig. 22:**
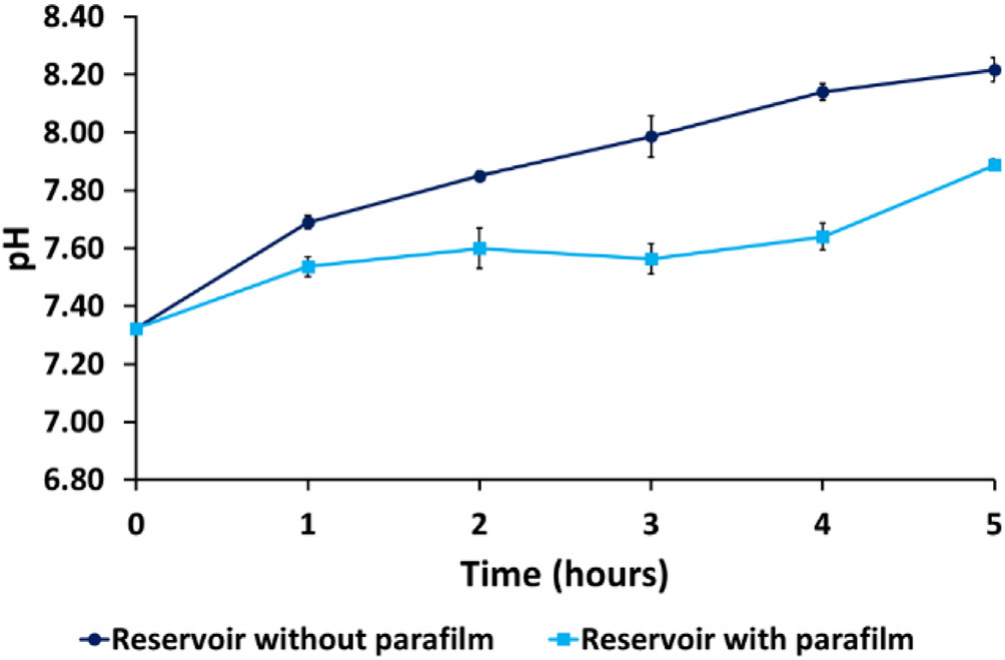
The comparison of the cell media pH evolution inside the reservoir with and without Parafilm. Error bar from standard deviation (N = 3).

Cell metabolism is greatly influenced by pH variations in the culture media. Prolonged exposure to sub-optimal pH significantly affects cell maturation, proliferation, and gene expression. Thus, keeping a neutral pH in the cell culture microenvironment is of critical importance. Most of the cell culture media are designed to maintain a constant pH around 7.4 under a 5% CO2 atmosphere inside an incubator. Since the carrier does not have any CO2 control, we investigated the effect of the atmospheric conditions on the medium pH when the carrier was moved out of the incubator. For the experiment, cell media was kept in the incubator in an Eppendorf tube with a membrane lid for 24 hr. Before taking out the samples, the Eppendorf tube was sealed by sandwiching Parafilm under the membrane lid to reduce gas exchange outside the incubator. The pH evolution was assessed over 5 h with triplicate measurements every hour using a pH meter (Seven Compact, Mettler Toledo). For comparison, the pH of the cell media contained in an Eppendorf tube without Parafilm sealing was tested over the full course of the experiment. The results are reported in Fig. 22.

The presence of the Parafilm resulted in a reduced gas exchange compared to the samples without the Parafilm. The samples that were not covered with Parafilm showed a steady pH increase throughout the entire experiment, whereas the samples that were protected with Parafilm reached a plateau around 7.6 after 1 hr and remained at this value for 3 hrs. After 3 hrs the pH increase of the sample without Parafilm was 2 times higher than the sample with the Parafilm.

The experiment was repeated with the Parafilm sealed reservoir mounted on the carrier and connected to the microfluidic chip with perfusion to replicate the conditions of the chip imaging. The pH obtained after 3 hrs was again 7.6, demonstrating that this is a reliable method to maintain pH at a physiologically relevant level even when intermittent perfusion was applied. The first three hours were chosen as the imaging window for the biological samples in the validation section.

### Relevant use case – cell proliferation with live stain on-chip

To demonstrate the use of our platform for long-term cell culture and repeated monitoring, including multiple incubator/microscope transfers, mouse brain endothelial cells were cultured in a glass/PDMS/glass microfluidic device for 10 days. Cell proliferation was assessed by nuclei counting at days 1, 3, 7 and 10.

**Fig. 23:**
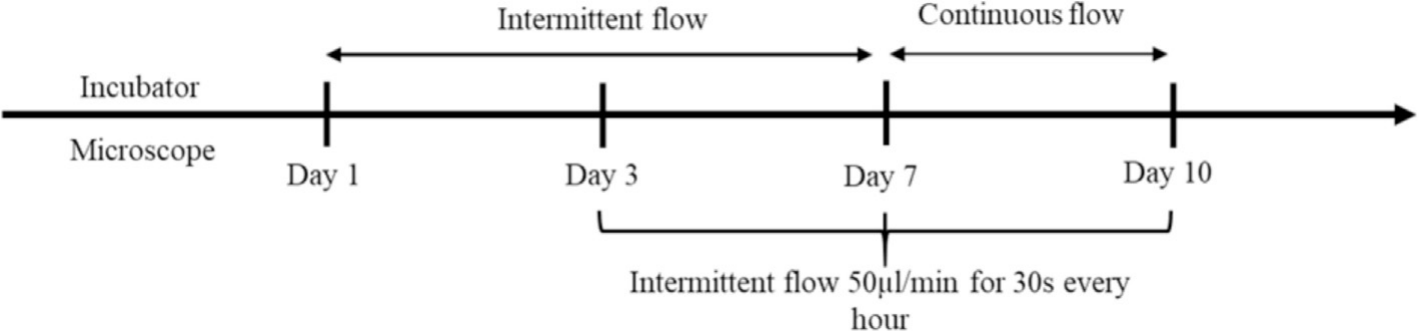
The protocol followed for the cell culture on the carrier both in the incubator and on the microscope. The imaging was performed at days 1, 3, 7 and 10, with cell media changed on days 1, 3 and 7.

For the experiment with the carrier, assembly and sterilisation followed the procedure described in the assembly section. The microfluidic chip was autoclaved before being maintained for one day in the incubator with cell media to allow the PDMS layer to equilibrate with the incubator atmosphere. The mouse cerebral microvascular endothelial cell line, bEnd.3 cells, at passage 7, were cultured in a flask in high glucose DMEM cell media (Gibco, Thermofisher, supplemented with GlutaMAX, 10% FBS and 2% penstrip). The cells were seeded in the microfluidic chip at a density of 10^6^ cells/ml and allowed to adhere under static conditions for 2 hrs after which the channels were flushed with fresh cell media to remove the unattached cells. Intermittent perfusion with cell media turnover (100 µl/min for 20 s, 0.8 dyne/cm^2^) was performed every 8 hrs until cells reached a confluent layer (after around 24 hrs). Then, the intermittent perfusion interval was changed from every 8 hrs to 1 hr until day 7. From day 7 and until the end of the experiment, the perfusion was operated in continuous flow (100 µl/min). During imaging, an intermittent flow rate of 50 µl/min for 30 s every hour was performed to prevent cell starvation and media acidification in the microfluidic channel. The flow rate pattern during the 10-day experiment is shown in Fig. 23.

As biological evaluation, two carriers were kept on the microscope stage for 3 hrs at each data point to mimic long imaging sessions while the imaging for two control carriers was minimised (less than 15 min) to reduce the sample time outside the incubator. The cell proliferation was compared to determine the biological response of 3 hr imaging sessions with heating and intermittent flow of the carrier against the cells that were only exposed to a very short time on the microscope stage, Fig. 25.

Cells were stained with a live nucleus fluorescent dye (Spy DNA, Spirochrome) by direct injection inside the reservoir with a final 1000× dilution. The fresh cell staining solution was added at each data point and subsequently washed with fresh media after cell imaging. No bubbles or contamination was observed due to the use of the carrier, although multiple reagent addition steps were performed. The system simplified the perfusion, staining and imaging process by eliminating cumbersome external perfusion equipment and enabling the microfluidic chip to be transferred between the incubator and microscope without any tubing disconnections. The combination of 1.5 ml reservoirs and cell media recirculation reduced the cell media consumption.

**Fig. 24:**
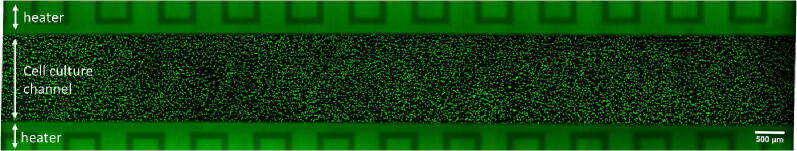
The overview of the available section for Imaging of the microfluidic channel. The cells were stained with live nucleus staining (green), day 3. The heater PCB substrate gives a fluorescent signal. The repetitive pattern on the heater represents the copper serpentine. (For interpretation of the references to colour in this figure legend, the reader is referred to the web version of this article.)

Cell imaging was performed with a Leica SP8 inverted laser scanning microscope with a 10× and 0.3 N.A. objective in the channel section overlapping the aperture of the carrier, Fig. 24.

**Fig. 25:**
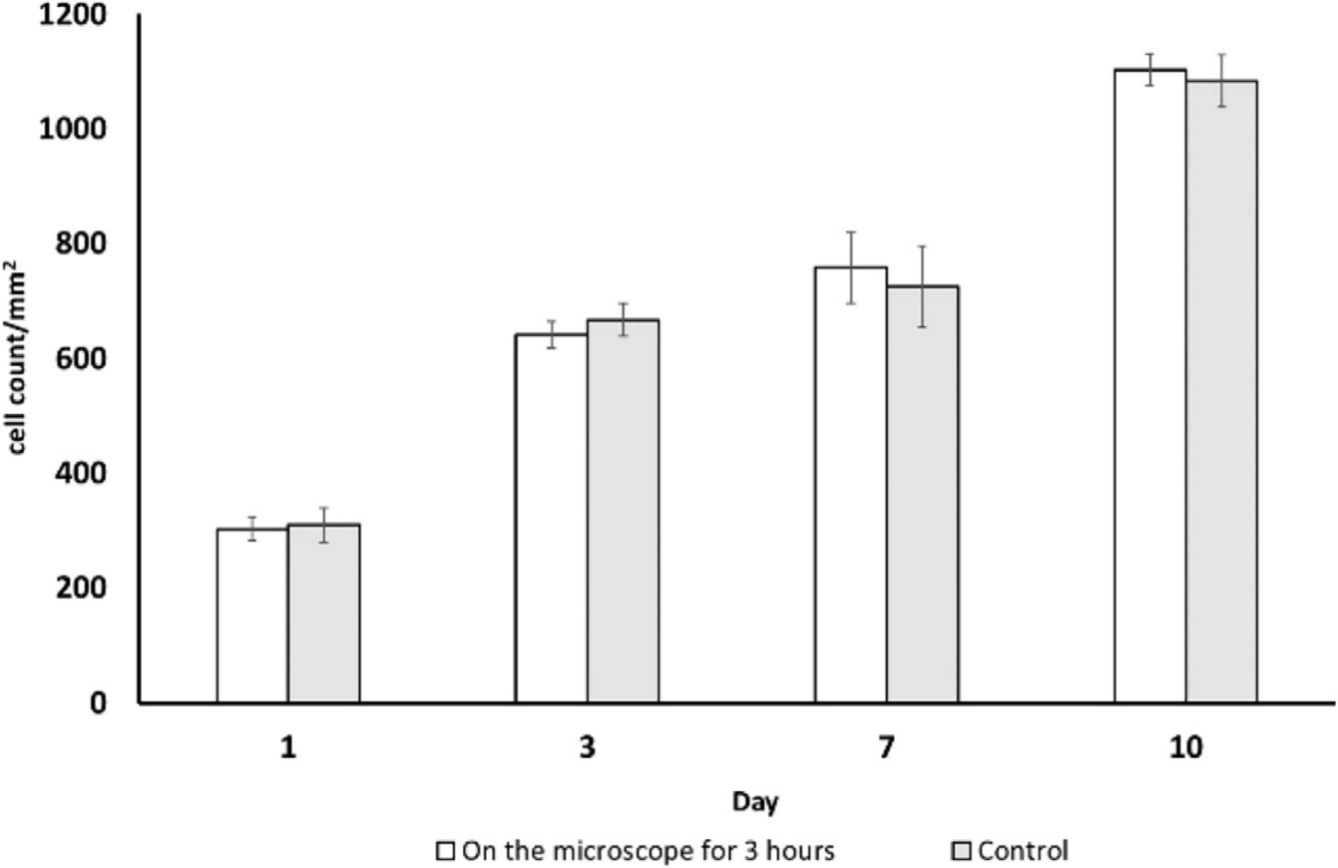
The cell proliferation of b.End3 cells after 1 day, 3 days, 5 days, and 10 days of culture. Error bar from standard deviation (N = 4).

The carrier design allowed easy access to the microfluidic chip channel in the sample with the microscope objective. The acquired images for the cell proliferation study were processed using Cell Profiler to automatically identify cell nuclei [20]. Cell proliferation over the 10-day culture is shown in Fig. 25.

The cell proliferation study showed a steady growth of b.End3 cells in the carrier over the 10-day experiment. Cells kept on the microscope stage for 3 h with carrier heating and periodic cell media perfusion displayed a proliferation rate comparable to the control. Cell proliferation increased between day 1 and day 3, where it reached a plateau until day 7. The continuous flow applied from day 7 resulted in an increase in cell proliferation until the experiment was stopped at day 10.

**Fig. 26:**
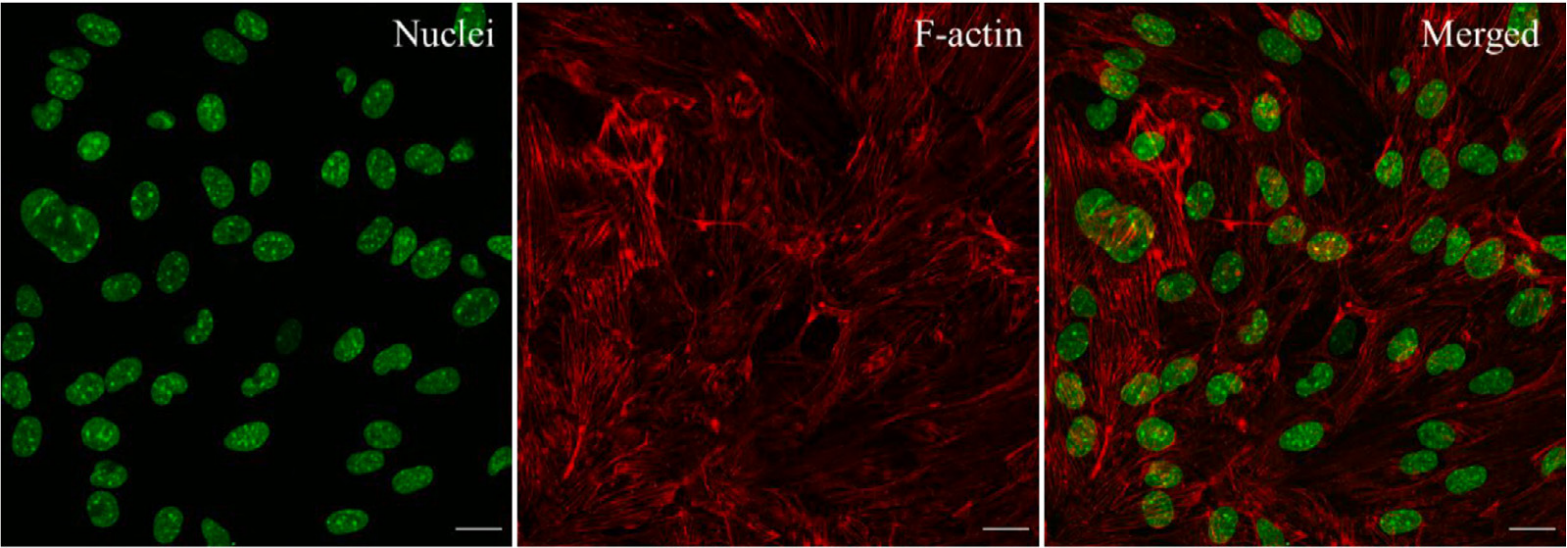
The confocal imaging of b.End3 cells. F-actin staining (red). Nuclei staining (green), day 10. Scale bar = 20 µm. (For interpretation of the references to colour in this figure legend, the reader is referred to the web version of this article.)

The ability to acquire images during cell media perfusion in the microfluidic chip on the microscope stage was investigated by imaging live nucleus and F-actin staining (1000× dilution, Spy F-actin, Spirochrome) with a 20× and 0.5 N.A. objective, Fig. 26.

The wide aperture of the carrier combined with the thinness of the heater (less than 50 µm) and chip lateral support (around 200 µm) allowed the image to be sampled with a working distance objective of around 1 mm.

**Fig. 27:**
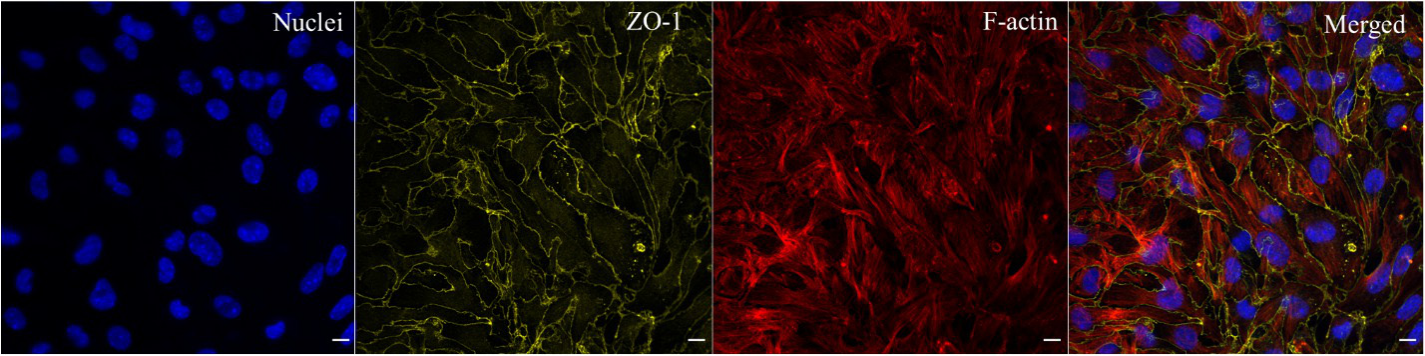
The confocal imaging of bEnd.3 cells. Nuclei staining (blue). ZO1 staining (yellow). F-actin staining (red). Scale bar = 10 µm. (For interpretation of the references to colour in this figure legend, the reader is referred to the web version of this article.)

After completing the cell culture, the microfluidic platform was removed from the carrier. The tight junction formation was then investigated by assessing the ZO-1 protein expression. Removal of the microfluidic chip was possible by first detaching the chip lock and then disconnecting the inlet and outlet tubing. All the reagents and washing buffers were injected by pipetting the solution into the microfluidic chip. The endothelial cells were fixed with 2% PFA solution for 10 min at room temperature after washing the respective channel three times with PBS. Subsequently, cell permeabilization was performed with a 0.05% Triton-X100 (Sigma-Aldrich) in PBS solution for 5 min at room temperature. After following the same washing step as used for the fixing solution, the sample was blocked for 120 min with 3% BSA (Thermofisher Scientific) and 0.2% IGEPAL (Sigma-Aldrich) at room temperature. Then, the primary antibody ZO-1 in 1:1 blocking solution and PBS (1:100 dilution, Thermofisher Scientific, 40-2300) was added. After overnight incubation at 4 °C, the sample was flushed three times with PBS. The secondary antibody (Abcam, ab150077) in blocking solution (1:200 dilution) was injected and incubated for 120 min at room temperature. After washing three times with PBS, the sample was incubated with Hoechst (1.5 µM, Thermofisher Scientific) and F-actin (1000× dilution, Spirochrome) solution for 30 min to stain the nuclei and the F-acting filaments, respectively. Finally, the solution was removed with three PBS washing steps and imaging was performed with a 25× and 0.95 N.A. objective, Fig. 27.

The b.End3 cells showed the formation of the tight junction after the completion of the culture, demonstrating the capability of more in-depth cell analysis after long-term cell culture in the presented platform.

## Conclusions

In this work, we have presented an integrated system that enables repeated transfers of microfluidic chips used for cell culture between an incubator and a microscope whilst maintaining a physiological temperature and periodic cell media turnover. The carrier integrates miniaturised piezo pumps that allow for a wide range of flow rates (here operated from 50 µl/min up to 1,000 µl/min) in continuous or intermittent modes. The system also includes integrated heaters made from copper that offer a stable and uniform temperature of 37 °C ± 0.5 °C outside the incubator. The compact set-up provides easy access to the reservoirs which simplifies sample collection or addition of reagents without the risk of introducing contaminants or air bubbles. Moreover, the recirculation of cell media combined with 1.5 ml reservoirs resulted in low reagent consumption. We demonstrate the successful culture of b.End3 cells that were regularly monitored with microscopy outside the incubator at days 1, 3, 7 and 10 for three hours at a time, with no effect on cell proliferation. The carrier represents a versatile tool for microfluidic users in need of a cost-effective platform offering a wide range of flow rates and different perfusion behaviours in a temperature-controlled environment for frequent microscopy monitoring.

## Human and animal rights

No humans or animals were harmed in this study.

## CRediT authorship contribution statement

**Federico Cantoni:** Conceptualization, Data curation, Formal analysis, Investigation, Methodology, Project administration, Validation, Visualization, Writing – original draft, Writing – review & editing. **Gabriel Werr:** Conceptualization, Formal analysis, Investigation, Methodology, Software, Validation, Visualization, Writing – review & editing. **Laurent Barbe:** Conceptualization, Project administration, Software, Supervision, Writing – review & editing. **Ana Maria Porras:** Methodology, Writing – review & editing. **Maria Tenje:** Conceptualization, Funding acquisition, Project administration, Supervision, Visualization, Writing – review & editing.

## Declaration of Competing Interest

The authors declare that they have no known competing financial interests or personal relationships that could have appeared to influence the work reported in this paper.
